# Design, Synthesis, Evaluation and Molecular Dynamics Simulation of Dengue Virus NS5-RdRp Inhibitors

**DOI:** 10.3390/ph16111625

**Published:** 2023-11-17

**Authors:** Keli Zong, Wei Li, Yijie Xu, Xu Zhao, Ruiyuan Cao, Hong Yan, Xingzhou Li

**Affiliations:** 1 Faculty of Environment and Life, Beijing University of Technology, 100 Pingleyuan, Beijing 100124, China; zongkeliya@sina.com; 2 Beijing Institute of Pharmacology and Toxicology, 27 Taiping Road, Beijing 100850, China; a_moon1096@163.com (W.L.); beilou2353@163.com (Y.X.); caoruiyuan@bmi.ac.cn (R.C.); 3 Department of Hepatology, Fifth Medical Center of Chinese PLA General Hospital, 100 West Fourth Ring Road, Beijing 100071, China; xuzhao080@outlook.com

**Keywords:** dengue virus, antiviral compounds, NS5-RdRp inhibitors, molecular docking

## Abstract

Dengue virus (DENV) is a major mosquito-borne human pathogen in tropical countries; however, there are currently no targeted antiviral treatments for DENV infection. Compounds **27** and **29** have been reported to be allosteric inhibitors of DENV RdRp with potent inhibitory effects. In this study, the structures of compounds **27** and **29** were optimized using computer-aided drug design (CADD) approaches. Nine novel compounds were synthesized based on rational considerations, including molecular docking scores, free energy of binding to receptor proteins, predicted Absorption, Distribution, Metabolism, Excretion, and Toxicity (ADMET) parameters, structural diversity, and feasibility of synthesis. Subsequently, the anti-DENV activity was assessed. In the cytopathic effect (CPE) assay conducted on BHK-21 cells using the DENV2 NGC strain, both SW-b and SW-d demonstrated comparable or superior activity against DENV2, with IC_50_ values of 3.58 ± 0.29 μM and 23.94 ± 1.00 μM, respectively, compared to that of compound **27** (IC_50_ = 19.67 ± 1.12 μM). Importantly, both SW-b and SW-d exhibited low cytotoxicity, with CC_50_ values of 24.65 μmol and 133.70 μmol, respectively, resulting in selectivity indices of 6.89 and 5.58, respectively. Furthermore, when compared to the positive control compound 3′-dATP (IC_50_ = 30.09 ± 8.26 μM), SW-b and SW-d displayed superior inhibitory activity in an enzyme inhibitory assay, with IC_50_ values of 11.54 ± 1.30 μM and 13.54 ± 0.32 μM, respectively. Molecular dynamics (MD) simulations elucidated the mode of action of SW-b and SW-d, highlighting their ability to enhance π–π packing interactions between benzene rings and residue W795 in the S1 fragment, compared to compounds **27** and **29**. Although the transacylsulphonamide fragment reduced the interaction between T794 and NH, it augmented the interaction between R729 and T794. In summary, our study underscores the potential of SW-b and SW-d as allosteric inhibitors targeting the DENV NS5 RdRp domain. However, further in vivo studies are warranted to assess their pharmacology and toxicity profiles.

## 1. Introduction

Dengue fever (DF), a mosquito-borne tropical ailment caused by the Dengue virus (DENV), is characterized by flu-like symptoms and may sporadically progress to severe and life-threatening complications, such as dengue hemorrhagic fever (DHF) or dengue shock syndrome (DSS) [1,2]. DF is the most widespread and rapidly increasing vector-borne disease (VBD) globally. It is now widespread in over 100 countries, affecting approximately 40% of the world’s population [3,4]. Each year, there are approximately 400 million infections, 500,000 severe cases of hospitalization, and a 2.5% mortality rate [5,6].

DENV, a member of the Flaviviridae family, is a single-stranded RNA (+ssRNA) virus [7]. Four serotypes of the virus (DENV-1, DENV-2, DENV-3, and DENV-4) can be distinguished [8,9,10], which differ in amino acid identity by 30–35% [11]. The treatment for dengue primarily relies on adjuvant therapy, as specific antiviral therapies are lacking [12]. There are also limitations to the use of a licensed dengue vaccine in naïve individuals not previously infected with DENV and in children under nine years of age [13,14]. An ideal dengue vaccine should possess cross-protective properties against all four serotypes, offer sustained efficacy, and ensure reliable safety. However, antibody-dependent enhancement (ADE) effects upon reinfection with other serotypes, and the absence of suitable animal models, make the development of an ideal dengue vaccine challenging [15,16]. Therefore, the development of safe and effective drugs for dengue treatment is urgently required.

The genome of DENV is approximately 10.7 kb in length and constitutes a single open reading frame (ORF) flanked by 5′ and 3′ untranslated regions (UTRs). The ORF of the genome encodes three structural proteins—capsid (C), premembrane/membrane (prM/M), and envelope (E)—along with seven nonstructural proteins (NS1, NS2A, NS2B, NS3, NS4A, NS4B, and NS5) [17,18,19]. Among these, NS5 acts as a methyltransferase and an RNA-dependent RNA polymerase (RdRp), both of which are essential for viral replication [20,21]. The DENV RdRp is responsible for both negative- and positive-stranded RNA synthesis during replication [22,23,24]. Since it lacks a mammalian counterpart and its sequence is conserved across all four serotypes with over 65% homology, it offers an attractive opportunity for the discovery of new antiviral drugs [25,26].

Interest in DENV NS5 has grown in recent years, and multiple RdRp inhibitors have been reported [27,28,29,30,31,32]. Notably, the Novartis Institute for Tropical Diseases has identified two promising allosteric inhibitors of DENV RdRp: 8-quinolyl sulfonamide (27) and 3-methoxybenzene ring (29) derivatives, demonstrating substantial activity, with IC_50_ values of 0.013–0.074 μM and 0.048–0.172 μM, respectively, against all clinically relevant dengue virus serotypes [9,33]. In the continuing pursuit of small molecular entities targeting DENV, we attempted to use compounds **27** and **29** as lead compounds to identify new anti-DENV compounds with higher activity. This article describes our efforts in this regard, shedding light on promising avenues for the development of future antiviral therapies and contributing to the fight against the dengue virus.

## 2. Results and Discussion

### 2.1. Compounds Design

#### 2.1.1. Binding Mode Analysis of Compounds **27** and **29**

**Figure 1:**
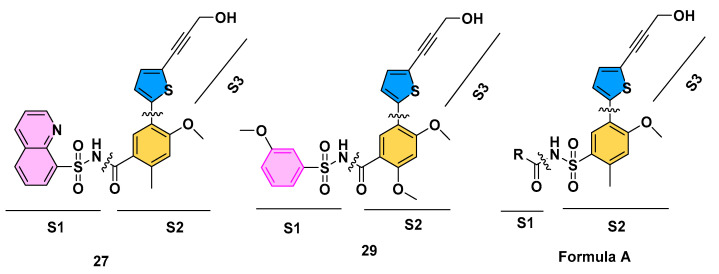
Compounds **27**, **29**, and Formula A.

**Figure 2:**
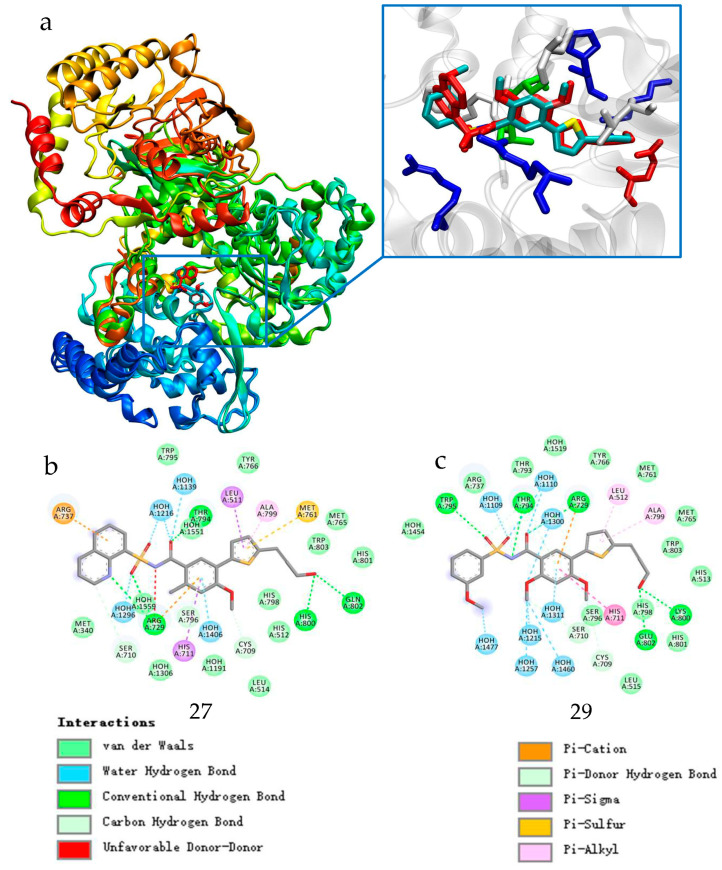
(**a**): Overlap of compound **27** (red) with the NS5-RdRp crystal complex (PDB:5JJR) and compound **29** (blue) with the NS5-RdRp (5K5M) crystal complex; (**b**): two-dimensional representation of the interaction analysis results of compound **27** with protein; (**c**): two-dimensional representation of the interaction analysis results of compound **29** with protein.

From a structural perspective, compounds **27** and **29** can be divided into three segments: *N*-aryl sulfonyl formamide fragment S1, thiophene-substituted benzene S2, and propargyl alcohol fragment S3, as shown in Figure 1. The three-dimensional crystal structures of compounds **27** and **29** with DENV RdRp protein complexes have been previously reported [29,33,34]. An analysis of the binding modes was conducted using the crystal structures of compounds **27** and **29** with DENV RdRp, as shown in Figure 2a. Upon comparing the binding modes, the S2 and S3 fragments of compounds **27** and **29** were found to form stable interactions with DENV NS5 RdRp, showing minimal variability. In contrast, the S1 fragment displayed a relatively high degree of variability in its binding to DENV NS5 RdRp. Specifically, the S1 fragments of compounds **27** and **29** adopted distinct conformations when binding to DENV NS5 RdRp.

It can be easily seen from the Figure 2b,c that the carbonyl group of S1 in compound **27** formed a hydrogen bond (HB) with the side chain of T794, and the sulfonyl group of the S1 moiety formed another HB with the backbone amide of R729. Furthermore, the amino group of the acyl sulfonamide dissociated into an amino anion and formed a salt bridge with the side-chain guanidine group of R729. The *N*-sulfonyl formamide moiety of compound **29** displayed an entirely different orientation. Its carbonyl moiety formed an HB with the side-chain guanidine of R729, and the sulfonyl moiety formed an HB with the backbone amide of W795. Additionally, the amino anion of *N*-sulfonyl formamide formed an HB with the side-chain hydroxyl group of T794. Despite their special differences, the “acyl-sulfonamide” moiety of S1 in both compounds **27** and **29** formed multiple strong HB interactions with DENV NS5 RdRp. However, the aryl moieties of S1 (the 8-hydroxyquinoline ring in compound **27** and methoxy-substituted phenol ring in compound **29**) extended into the solvent with distinct orientations, without forming strong interactions with RdRp. In addition, a substantial cavity was observed near the binding site of S1 from Figure 2a.

Therefore, we envisioned that retaining the S2 and S3 fragments along with the “sulfonyl formamide” portion of the S1 fragment, which have strong interactions with RdRp, and replacing the aryl moiety of S1, which has greater conformational flexibility and weaker protein binding, with a larger group that occupies the cavity near the S1 binding site, could increase the affinity of the compound for NS5-RdRp. To facilitate synthesis of the designed compounds, the positions of the sulfonyl and carbonyl groups of the *N*-sulfonyl formamide were reversed (converted to *N*-acylsulfonamide), since such acylsulfonamide compounds have been reported to still have good inhibitory activity against DENV NS5-RdRp [9]. Based on this concept, we designed compounds with Formula A, as shown in Figure 1 and attempted to identify suitable substituents to replace the S1 aryl group through a combination of virtual compound library construction and docking-based virtual screening.

#### 2.1.2. Construction of Virtual Compound Library

To construct a focused virtual library of *N*-acylsulfonamide derivatives, diverse carboxylate moieties were linked to the sulfonamide of Formula A using the Enumerate Library by Reaction protocol (Discovery Studio 3.0). This allowed exploration of the chemical space involving carboxyl substitution on the sulfonamide N. The building blocks were derived from a small-molecule carboxylic acid fragment library (Fragment Library_12126) consisting of 1824 compounds. Subsequently, the LigPrep module was employed to apply a force field OPLS3e, generating potential ionization states and tautomers at pH 7.0 ± 2.0, along with multiple conformers for the resulting 1824 molecules.

#### 2.1.3. Virtual Screening and Selection of Target Compounds

##### Receptor Selection

The refined coordinates for the full-length co-crystal structures of DENV3 RdRp with compounds **27** and **29,** as well as those for the DENV2-NGC RdRp co-crystal with compound **29**, have been deposited in the Protein Data Bank (PDB) under accession codes 5I3P, 5I3Q, and 5K5M, respectively. The crystal structures of the full-length DENV3 NS5 protein, containing compounds **27** and **29,** are also available as PDB entries 5JJS and 5JJR [33]. Among the several subtypes of DENV, we chose the DENV2 RdRp protein complex containing compounds **27** and **29** as the docking receptors because DENV2 is the subtype most likely to cause severe disease. Among the reported DENV2 RdRp-ligand complexes, we chose PDB 5K5M (full-length DENV2 RdRp co-crystals with compound **29**) as the acceptor protein for virtual screening. This selection was based on its superior resolution (resolution: 2.01 Å) and the presence of a suitably sized cavity adjacent to the S1-occupied site [33].

##### Verification of the Docking Method Preparation

**Figure 3:**
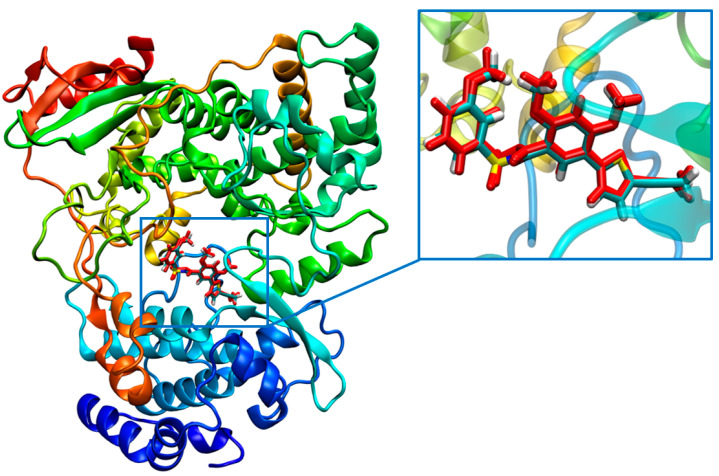
Comparison between the highest ranked pose of compound **29** (cyan) and the original pose (red) in the crystal structure. The original pose was presented as blue stick, the predicted top-ranked pose was presented as red stick.

To investigate the applicability and reliability of the docking method, we first attempted to redock the original ligand (compound **29**) to its binding site in the crystal structure using the Glide module. DENV2 RdRp combined with compound **29** was prepared using the Protein Preparation Wizard module, and the glide grid for docking was generated using the Receptor Grid Generation module, as described in the experimental section. Compound **29** was extracted from the proteins and prepared using the LigPrep module. The glide docking of compound **29** was performed with XP precision, using the aforementioned grid files. The ligand was set to be flexible with default parameters, and post-docking minimization was conducted. We found that the root-mean-square deviation (RMSD) value between the highest-ranked pose (XP GScore of −11.995 kcal/mol) and the original pose was 2.54 Å. Additionally, it can be easily seen from Figure 3 that compound **29** and original ligand were in similar stacking maps. These results suggesting that this docking procedure accurately predicted the binding pose of the true substrate.

##### Virtual Screening, ADMET Prediction, Binding Free Energy Calculation, and Compound Selection

The ligand poses of the 1824 molecules in the virtual compound library, generated by LigPrep, were subjected to ligand docking using an extra-precision (XP) ligand flexible docking process. This was performed to identify molecules with better adaptability to the dengue virus NS5-RdRp. The Glide grid file and docking parameters used during docking were the same as those described in the docking verification section. The docking results were sorted based on the docking scores, where a more negative value indicates stronger binding of the compound to the protein. Docking scoring provides a quick estimate of binding affinity; however, docking scoring methods do not account for solvent effects, which may affect accuracy. To address this, binding free energy calculations were performed using the MMGBSA method. This method calculates the free energy difference between the drug and target molecules, incorporating solvent effects, thus providing more accurate results. Consequently, docking complexes of these top-ranking compounds with an XP GScore below −9.0 were subjected to Prime MM-GBSA calculations to determine their binding free energies (Kcal/mol) with the NS5 RdRp subunit. Considering the Docking Score and binding free energy, compounds with a Docking Score lower than −10 and binding free energy (∆Gbind) lower than −80 kcal/mol were selected for initial selection.

In addition to examining the docking score ∆Gbind and structural diversity, we also evaluated the ADME properties of each compound, with a focus on solubility, absorbability, and membrane permeability, as these properties significantly affect activities. The ADMET protocol in Discovery Studio 3.0 (DS3.0) was used to calculate solubility and absorbability predictors. Solubility was evaluated using the “ADMET Aqueous Solubility Level” predictor, and absorbability was evaluated using the “ADMET Absorption Level” predictor. Membrane permeability was evaluated using the QPPCaco parameter, which was predicted using the QikProp protocol in Schrödinger (2020). Compounds with ADMET absorption levels of 0 (good), 1 (moderate), or 2 (low absorption), ADMET Aqueous Solubility levels of 2 (yes, low), 3 (yes, good), or 4 (yes, optimal), and QPPCaco values higher than 80 nm/s were chosen for further consideration. Considering their synthesizability, nine compounds were selected for synthesis. The structures, docking scores, ADMET parameter, and MM-GBSA ΔG-bind values of these selected target compounds are presented in Table 1.

### 2.2. Synthesis

**Scheme 1:**
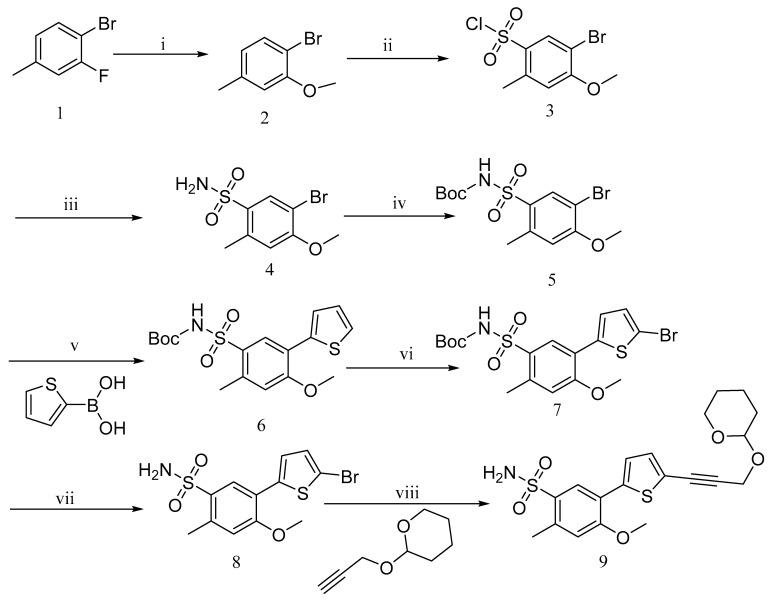
Synthetic outline for the preparation of compound **9**. Reagents and conditions: (i) MeOH, MeONa, 95 °C; (ii) ClSO_2_OH, CH_2_Cl_2_, 80 °C; (iii) MeOH, NH_3_·H_2_O, room temperature (rt), 40%; (iv) MeOH, (Boc)_2_O, TEA, DMAP, 35 °C, 72.2%; (v) 1,2-dimethoxyethane, H_2_O, 2-Thiopheneboronic acid, Na_2_CO_3_, Bis(triphenylphosphine)palladium(II) chloride, N_2,_ 80 °C, 45%; (vi) DMF, NBS, BPO, 90 °C, 74.5%; (vii) CH_2_Cl_2_, TFA, rt, 96%; (viii) 1,4-Dioxane, 2-prop-2-ynoxyoxane, CuI, TEA, Pd(dppf)Cl_2_·CH_2_Cl_2_, N_2_, 90 °C, 56%.

Compound SW was obtained by coupling two key fragments: the benzenesulfonamide fragment (**9**) and the carboxylic acid fragment (**25**). The key intermediate, benzenesulfonamide (**9**), was synthesized in multiple steps according to the literature’s procedures (Scheme 1) [9,35,36]. 4-Bromo-3-methoxy-toluene (**2**) was obtained via nucleophilic displacement of 4-bromo-3-fluorotoluene (**1**) with sodium methoxide [37]. The 5-position of compound (**2**) was chlorosulfonated using chlorosulfonic acid, and then reacted with aqueous ammonia to yield the sulfonamide derivative 5-bromo-4-methoxy-2-methylbenzenesulfonamide (**4**) [9]. The amino group was protected using (Boc)_2_O in the presence of triethylamine and DMAP, resulting in tert-butyl ((5-bromo-4-methoxy-2-methylphenyl)sulfonyl)carbamate (**5**). The Suzuki– Miyaura cross-coupling reaction of compound **6** with thiophene boronic acid produced ((4-methoxy-2-methyl-5-(thiophene)yl)phenyl)sulfonyl) tert-butyl carbamate (**6**) [9]. The resulting compound **7** was subjected to halogenation of the thiophene ring using benzoyl peroxide and NBS, and the protecting group attached to the amino group was removed using trifluoroacetic acid to obtain 5-(5-bromothiophen-2-yl)-4-methoxy-2-methylbenzenesulfonamide (**8**) [9,38]. Under the catalysis of cuprous iodide, triethylamine, and the 1,1′-bis(diphenylphosphino)ferrocene-dichloropalladium(II) dichloromethane complex, compound **8** was cross-coupled with propargyloxytetrahydropyran to obtain the key intermediate **9** [39]. This intermediate was used directly in the subsequent step, with an overall yield of approximately 5%.

**Scheme 2:**
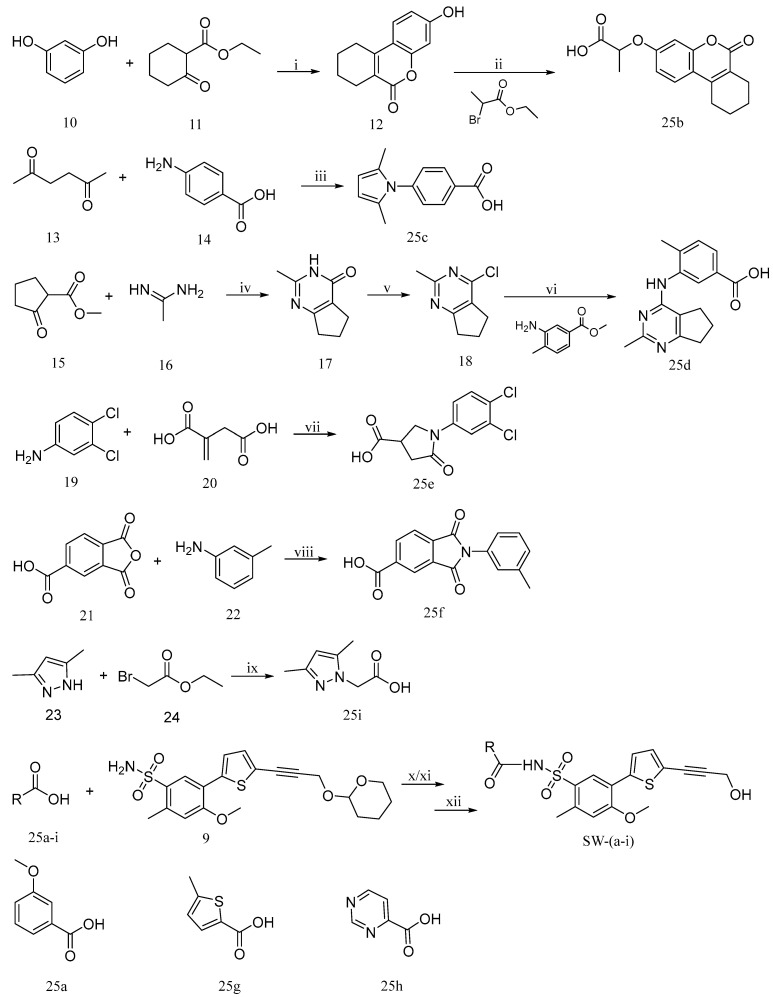
Synthetic outline for the preparation of compounds **SW-(a-i)**. Reagents and conditions: (i) CH_3_SO_3_H, aluminium oxide, Ethyl 2-cyclohexanonecarboxylate, EtOH, room temperature (rt), 77%; (ii) DMF, 2-bromopropanoic acid ethyl ester, K_2_CO_3_, 80 °C, 80%; (iii) CH_3_COOH, 95 °C, 71%; (iv) KTB, tert-Butanol; (v) POCl_3_; (vi) IPA, HCl, Methyl 3-amino-4-methylbenzoate, 95 °C, THF, H_2_O, LiOH, 58%; (vii) 100 °C, 89%; (viii) CH_3_COOH, 130 °C, 70%; (ix) K_2_CO_3_, CH_3_COCH_3_, H_2_O, NaOH, reflux, 80%, (x) CH_2_Cl_2_, HATU, DIPEA, 35 °C; (xi) CH_2_Cl_2_, EDCI, DMAP, 35 °C; (xii) MeOH, CH_2_Cl_2_, aq NH_4_Cl, aq citric acid, 45 °C.

Carboxylic acids, such as 3-methoxybenzoic acid (**25a**), 5-methyl-2-thiophenecarboxylic acid (**25g**), and 4-pyrimidinecarboxylic acid (**25h**), are commercially available. Carboxylic acid (**25b**–**f**) and **25i** were synthesized, as shown in Scheme 2. Briefly, commercially available resorcinol **10** and ethyl 2-oxocyclohexanecarboxylate **11** were cyclized in the presence of methanesulfonic acid to produce the corresponding coumarin analog **12**, which was subsequently nucleophilized with bromide in DMF, followed by deprotection with sodium hydroxide to furnish **25b** [40]. Compound **25c** was synthesized by a Paal–Knorr reaction between 2,5-Hexanedione **13** and 4-aminobenzoic acid **14** formate in the presence of acetic acid [41]. The cyclization of methyl 2-cyclopentanonecarboxylate 15 and ethanimidamide **16** generated the pyrimidine derivative **17** in the presence of potassium tert-butoxide. Halogenation of the pyrimidine ring **17** in the presence of phosphorus oxychloride (POCl_3_) provided compound **18**, which underwent nucleophilic reaction with lithium 3-amino-4-methylbenzoate, followed by deprotection with hydroxide·water (LiOH·H_2_O) to give **25d** [42]. A mixture of itaconic acid **20** and 3,4-dichloroaniline **19** was melted by heating, and the product **25e** was liberated with hydrochloric acid [43]. The reaction of trimellitic anhydride **21** with trimethylaniline **22** under acidic conditions yielded compound **25f** [44]. Finally, condensation of 3,5-dimethylpyrazole **23** with ethyl bromoacetate **24**, followed by hydrolysis with NaOH, yielded **25i** [45].

Using 1-ethyl-3-(3-dimethylpropyl) carbodiimide (EDC)/1-hydroxybenzotriazole (HOBT) or 1-ethyl-3-(3-dimethylpropyl) base carbodiimide (EDC)/2-(7-azabenzotriazol-1-yl)-*N*, *N*, *N*’, *N*’-tetramethyluronium hexafluorophosphate (HATU) as a condensing agent, benzenesulfonamide (**9**) condensed with various carboxylic acids (**25**), followed by deprotection of tetrahydropyran with saturated ammonium chloride solution, afforded the title compounds **SW-(a-i)**, as shown in Scheme 2, and the overall yield was around 50%. All compounds were characterized by ^1^H NMR, ^13^C NMR, and mass spectrometry. HPLC detection confirmed that the purity of each compound was greater than 95%.

### 2.3. Biological Evaluation

#### 2.3.1. Cytopathic Effect (CPE) Inhibition Assay and Cytotoxicity Assay

The antiviral activities of the nine synthesized compounds against DENV were assessed using the DENV2 NGC strain and BHK-21 cells via CPE inhibition assays. **BCX4430**, **NITD008**, and **27** were used as positive controls (Figure S2). Five compounds **(SW-(a-d), SW-f)** were found to protect cells from DENV virus-induced CPEs, with IC_50_ (the concentration for 50% of maximal inhibitory) values ranging from 3.58 μM to 57.60 μM at non-cytotoxic concentrations, as shown in Table 2. Compound **SW-b** exhibited the highest DENV inhibitory activity with an IC_50_ value of 3.58 ± 0.29 μM. Compound **SW-d** also demonstrated good DENV inhibitory activity, with an IC_50_ value of 23.94 ± 1.00 μM. Cytotoxicity of these compounds was determined in MDCK cells, where the cell cytotoxicity (CC_50_) of **SW-b** and **SW-d** compounds was 24.65 µM and 133.70 µM, respectively (Table 2). The selectivity index (SI) values of SW-b and SW-d ranged from 5.58 to 6.89, as determined by the ratio of CC_50_ to IC_50_. Both the activity and SI of compound **SW-b** were higher than those of the positive control compounds **NITD008** (IC_50_ = 41.59 ± 2.95 μM, SI > 1.29) and **BCX4430** (IC_50_ = 15.45 ± 5.54 μM, SI > 2.40). Its activity was also higher than that of compound **27** (19.67 ± 1.12μM). The activity of compound SW-d was higher than that of **BCX4430** and lower than that of **NITD008**; however, its SI was higher than those of **BCX4430** and **NITD008**.

#### 2.3.2. DENV Biochemical Enzyme Assay

The inhibitory activities of compounds **SW-b** and **SW-d** against DENV-2 RdRp were assessed using in vitro enzyme inhibition assays. Compounds **3′-dATP, BCX4430**, and **NITD008** were used as controls. Of the tested compounds, SW-b inhibited the activity of DENV2 RdRp with an IC_50_ of 11.54 ± 1.30 μM, while the IC_50_ value of SW-d against DENV2 RdRp was 13.54 ± 0.32 μM. The positive control compound **3′-dATP** also showed notable dengue RdRp inhibitory activity, with an IC_50_ of 30.09 ± 8.26 μM. It can be seen from Table 3 that the other two control compounds, **BCX4430** and **NITD008**, did not inhibit the DENV2 RdRp. This was reasonable, because these two compounds are nucleoside antiviral compounds that do not possess DENV2 RdRp inhibitory activity and need to be converted into their triphosphate form within cells to exert their RdRp inhibitory activities. This confirmed the reliability of the DENV2 RdRp inhibitory activity assay. Compounds **SW-b** and **SW-d** showed notable inhibitory activities against DENV2, with IC_50_ of 11.54 ± 1.30 and 13.54 ± 0.32 μM, respectively.

In summary, through evaluation of the nine compounds we designed and synthesized, we found that two compounds, **SW-b** and **SW-d**, show clear activity against DENV2 at the cellular level and obvious inhibitory effects on the RdRp activity of DENV2. The activity and selectivity index of compound **SW-b** were better than those of the known positive control compounds **BCX4430** and **NITD008**, as well as the lead compound **27**. These results suggest that compounds **SW-b** and **SW-d** specifically target DENV-RdRp and exert their anti-DENV-2 activity by inhibiting its enzymatic function.

### 2.4. Molecular Dynamics (MD) Simulation and Analysis

To elucidate the binding modes of compounds SW-b and SW-d to DENV NS5, we compared them with those of compounds **27** and **29**, bound to DENV NS5 using MD simulations conducted with Desmond [46]. Given the potential influence of chirality on small molecule–protein interactions, we conducted MD simulations considering two enantiomers of compound **SW-b**: **S-SW-b** and **R-SW-b**, even though compound **SW-b** was bioassayed as a racemate (Figure S1). To strike a balance between simulation accuracy and computational efficiency, the MD simulations had varying durations, ranging from 200–500 ns, with each run repeated three times to ensure consistent results. The duration depended on the structural stability of the protein–ligand complex.

An essential Indicator for assessing the simulated stability of protein–ligand complexes is the variation trend in the root-mean-square deviation (RMSD) values of proteins, and Figure S3. illustrates the RMSD of the DENV NS5 protein Cα atoms and the ligand RMSD fit to protein (Lig fit Prot) during the MD simulations. The protein RMSD trajectory is shown in blue with values on the left Y-axis (Å), while the ligand RMSD trajectory is shown in red with values on the right Y-axis in (Å).

The results showed that the RMSD of DENV NS5 protein *Cα* atoms tended to stabilize during the simulation of several complexes. Notably, there were no instances in which the Lig fit Prot value significantly exceeded the corresponding Cα RMSD value. This confirms the reliability of the MD simulation results, indicating that the ligands did not significantly deviate from their initial binding sites. Starting from the initial frames of the crystal complexes of DENV NS5-27 and DENV NS5-29, the protein Cα atoms and ligands reached stability quickly after 25 ns, indicating highly stable crystal structures. Regarding the DENV NS5-S-SW-b docking complex, the RMSD values of protein Cα atoms and Lig fit Prot reached stability at 10 ns. Although the RMSD value of protein Cα atoms increased slightly from 100 ns to 200 ns, it subsequently regained restability after 200 ns. For the DENV NS5-R-SW-b docking complex, the RMSD values of the protein Cα atom and the Lig fit Prot changed simultaneously. After 220 ns, they stabilized and fluctuated at approximately 1.5 Å. For the DENV NS5-SW-d docking complex, the RMSD values of the protein Cα atoms reached stability at approximately 20 ns, while the RMSD values for Lig fit Prot were less stable. After simulating for 280 ns, both the RMSD values of the protein Cα atom and Lig fit Prot reached stability. Nevertheless, after conducting MD simulations lasting between 300 ns and 500 ns, both systems achieved a state of relative stability. This finding underscores their fundamental structural reliability.

**Figure 4:**
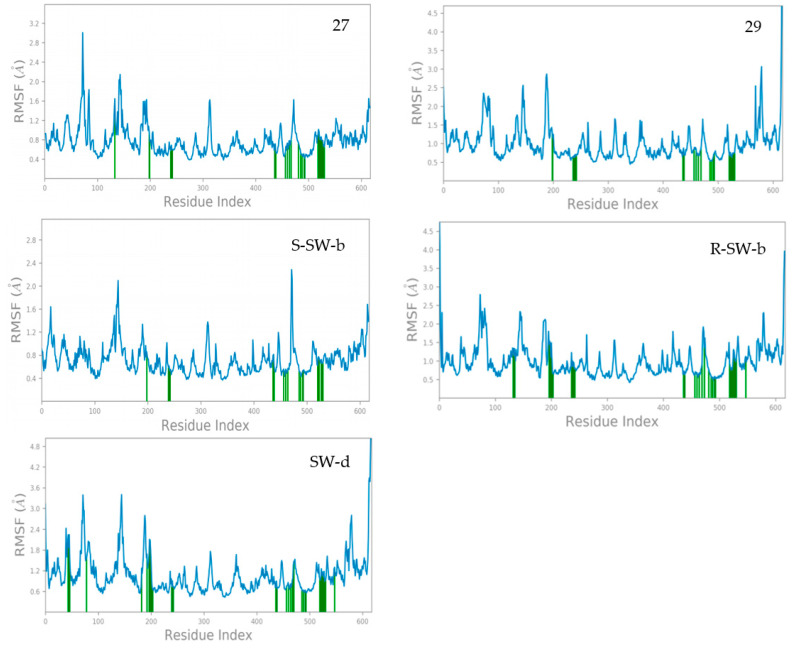
The RMSF of NS5 protein amino acid residues in MD simulations.

The root-mean-square fluctuation (RMSF) results of the amino acid residues in the DENV NS5 complex with compounds **27**, **29**, **SW-b** and **SW-d** during the MD simulations are shown in Figure 4. The RMSF reflects the flexibility of amino acid residues; a greater fluctuation in RMSF indicates the higher flexibility of the corresponding amino acid residues [47]. Protein residues interacting with the ligand are marked with green vertical bars. The RMSF fluctuations are useful for determining the stability of protein binding to small molecules.

It was observed that the crystal structure of the DENV NS5-27 complex (PDB: 5K5M) exhibited high stability. With the exception of the *N*-terminal and *C*-terminal regions of the protein and some amino acid residues, most amino acid residues had RMSF values below 2.0 Å. Specifically, the RMSF values of amino acid residues directly associated with compound **27** were basically less than 1.2 Å. In contrast, the crystal structure of the DENV NS5-29 complex was less stable compared to the DENV NS5-27 complex. Most amino acid residues had RMSF values below 3.0 Å, except for those located at the *N*-terminal and *C*-terminal regions of the protein. Furthermore, the RMSF values of amino acid residues directly interacting with compound **29** were basically less than 1.0 Å. In general, the complex structure of DENV NS5 with these two small-molecule inhibitors was stable. Compared with compounds **27** and **29**, compound **S-SW-b** demonstrated greater stability within the DENV NS5 protein. The RMSF values of the amino acid residues in the DENV NS5-S-SW-b complex basically did not exceed 2.0 Å, and the RMSF value of the amino acid residues directly interacting with compound **S-SW-b** was basically less than 0.8 Å. The stability of compound **R-SW-b** in the DENV NS5 protein was lower than that of compound **S-SW-b** but comparable to that of compound **29**. The RMSF values of amino acid residues in the DENV NS5-R-SW-b complex reached up to 2.5 Å, whereas the RMSF values of amino acid residues directly interacting with compound R-SW-b was basically less than 1.5 Å. Compared with the DENV NS5-27, **29**, and **SW-b** complexes, the stability of the DENV NS5-SW-d complex was slightly less pronounced. While a few amino acid residues exhibited RMSF values slightly exceeding 3.0 Å, the RMSF value of amino acid residues directly interacting with compound **SW-d** was approximately 1.8 Å. Overall, the complex formed between DENV NS5 and compound **SW-d** demonstrated a high degree of stability.

**Figure 5:**
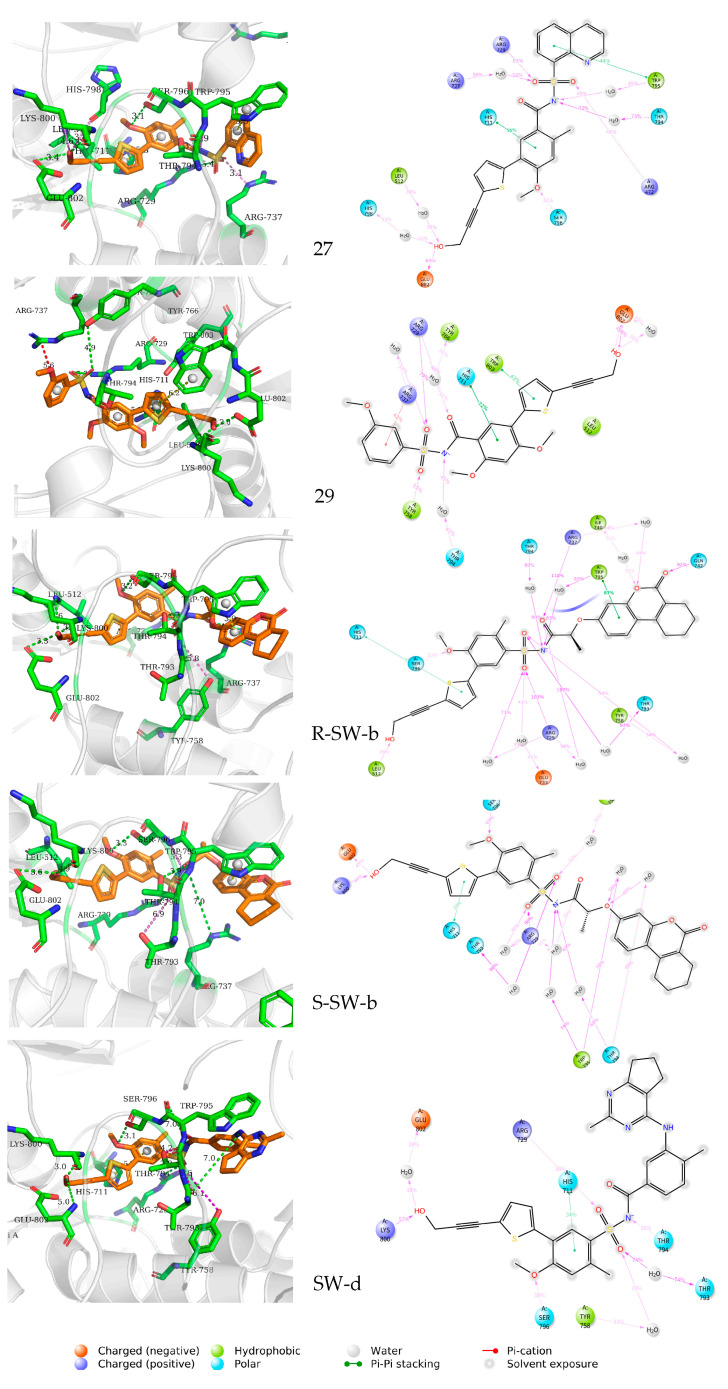
Two-dimensional representation and three-dimensional summary of the interaction analysis results of the NS5–ligand complexes (PDB: 5K5M). π–π stacking interactions are shown as yellow dashed lines, intermolecular hydrogen bonds as green dashed lines, and salt bridges as magenta dashed lines.

**Figure 6:**
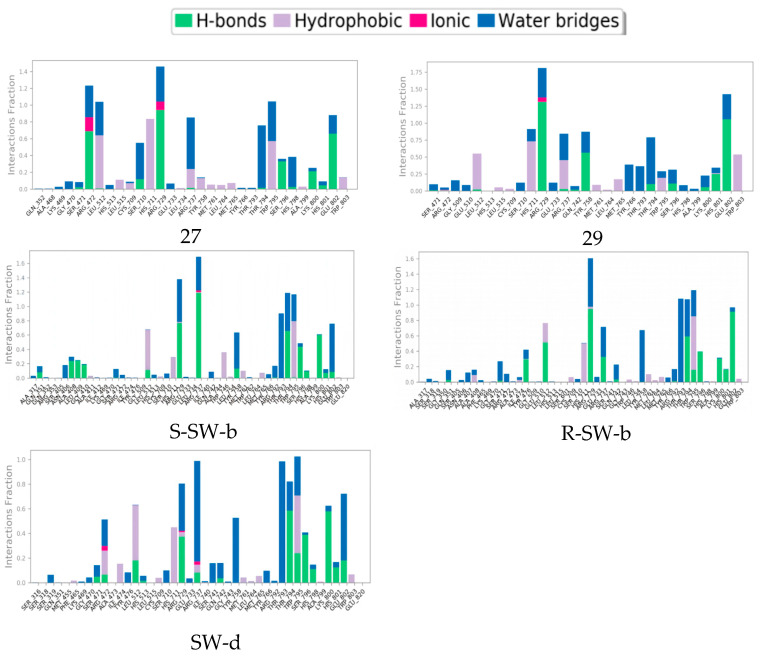
Interaction fraction summary of NS5–ligand contacts, normalized for total simulation time. Interaction fraction values over 1.0 indicate that a residue has multiple contact routes for interacting with the ligand.

The mode of action of the NS5–ligand complex was further resolved by protein– ligand contact and protein–ligand interaction scores. Protein–ligand contacts (or ‘interactions’) are categorized into four types: hydrogen bonds (HB), hydrophobic contacts (HP), ionic bridges, and water bridges (WB). Each interaction type contains more specific subtypes; for example, HP falls into three subtypes: ion–pi interactions (ion–pi), pi–pi stacking (π–π), and non-specific hydrophobic interactions (ns HP). A 2D summary of the protein–ligand contact analysis results for NS5 binding with compounds **27**, **29**, **S-SW-b**, **R-SW-b**, and **SW-d** is displayed (Figure 5). This can be visualized more intuitively in stick mode (Figure 6). Protein–ligand interactions, such as HB, WB, ion-pi interactions, and π–π stacking, which occurred with a probability of over 30% during the simulation, are displayed. A standardized stacked bar graph presents the protein–ligand interaction fractions of the four possible types of bond interactions (HB, HP, ionic, and WB) for NS5 binding with compounds **27**, **29**, **SW-b**, and **SW-d**. The interaction fraction of the protein with the corresponding residues is represented by a standardized stacked bar graph; for example, a value of 0.7 means that a specific interaction is maintained for 70% of the simulation time. Values above 1.0 are possible because certain protein residues may form multiple contacts of the same subtype with the ligand. A timeline representation of the interactions and contacts (hydrogen bonds and hydrophobic, ionic, and water bridges) is shown (Figure 6). The top panel shows the total number of specific contacts between the protein and ligand throughout the trajectory. The bottom panel shows the residues that interacted with the ligand in each trajectory frame. Some residues made more than one specific contact with the ligand, which is represented by the darker orange shade, according to the scale to the right of the plot.

In silico studies showed that the binding patterns of the S1 fragments of compounds **SW-b** and **SW-d** were different. In compound **SW-b**, the reverse acylsulfonamide amide moiety formed HB with the side chain of R737. Additionally, in compound **R-SW-b**, the propargyl alcohol hydroxyl moiety of S3 fragments formed HB with the side chain of L512. Compound **SW-d** mainly formed WB on the side chain R737 (Figure 4). During the MD simulations (Figure 5), **S-SW-b** formed hydrogen bonds with Q351, A408, L409, G410, S471, L512, R729, R737, Y758, R792, T794, W795, S796, H798, K800, H801, and E802. Of these, R729, R737, T794, and K800 had interaction fractions > 0.6. **R-SW-b** formed conventional hydrogen bonds with Q351, S471, Y476, L512, R729, R737, Q742, R792, T794, W795, S796, K800, H801, and E802. Of these, R729, T794, and E802 had interaction fractions > 0.6. **SW-d** formed conventional hydrogen bonds with S471, R472, L512, H513, R729, R737, Q742, T794, W795, S796, H798, K800, H801, and E802. Of these, T794 and K800 had interaction fractions > 0.6. Comparison with the protein–ligand contacts of compounds **27** and **29** shows that Compound **27** formed conventional hydrogen bonds with S471, R472, L512, S710, R729, R737, T794, S796, H798, K800, H801, and E802. Of these, R472, R729, and E802 had interaction fractions > 0.6. Compound **29** formed conventional hydrogen bonds with S471, R472, L512, R729, R737, Q742, Y758, T794, S796, K800, H801, and E802. Of these, R729 and E802 had interaction fractions > 0.6. Compounds **SW-b** and **SW-d** increased protein–ligand contact compared to compounds **27** and **29**. In addition, the benzene ring of the S1 fragments of **SW-b** and **SW-d** showed π–π stacking with W795. Compared with compounds **27** and **29**, the reverse acylsulfonamide fragment reduced the WB between T794 and NH but increased the WB between the sulfonyl group and R729 and T794. These findings suggest that compounds **SW-b** and **SW-d** possess similar or improved stabilities compared to compounds **27** and **29**.

**Figure 7:**
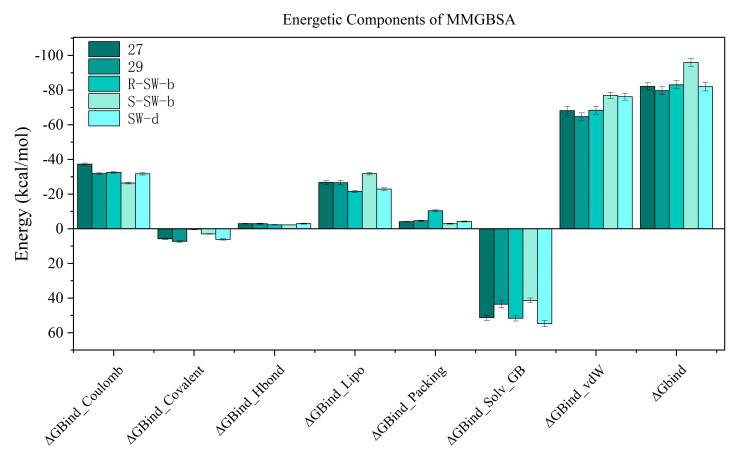
MM−GBSA energies of molecular simulation trajectory for compounds **27**, **29**, **R−SW−b**, **S−SW−b**, and **SW−d**.

The binding free energy profiles of the DENV NS5 protein complexes were evaluated using the MM/PBSA method. The estimated binding free energy of the DENV NS5 protein with compounds **27**, **29**, **R-SW-b**, **S-SW-b**, and **SW-d** were −82.138 kcal/mol, −79.716 kcal/mol, −83.090 kcal/mol −95.966 kcal/mol, and −82.003 kcal/mol, respectively, while S-**SW-b** was the best one among these compounds, confirming that it had a strong affinity. Additionally, the free energy of the protein–ligand complexes was composed of multiple components of energy (van der Waals, Coulomb, Covalent and Hbond, solvation, and Packing); solvation Lipo and van der Waals force was the major contributor to the total binding free energy (Figure 7). It was noteworthy that, compared to other compounds, a substantial reduction in the number of solvation and Lipo energy in **S-SW-b** can be observed, which may be a reason why it has the lowest total binding free energy.

In conclusion, our simulation results were consistent with the results of the biological activity detection. Among this group of compounds, the DENV NS5-SW-b complex demonstrated the highest stability, and compound SW-b exhibited the highest enzyme inhibitory activity. Conversely, the stability of the DENV NS5-SW-d complex was notably lower, and the corresponding compound, **SW-d**, displayed reduced enzyme inhibitory activity compared to compounds **27** and **SW-b**. This further validates the reliability of the simulation results. Moreover, our simulation results also indicated that the activity of compound **S-SW-b** should surpass that of its enantiomer **R-SW-b**.

## 3. Materials and Methods

### 3.1. Chemistry

#### General Chemistry Information

All materials and reagents employed in this study were of the highest commercially available grade and were used without further purification. Analytical thin-layer chromatography (TLC) was performed using Energy Chemical Silica Gel HF254 pre-coated plates (Merck, Kenilworth, NJ, USA). Flash column chromatography was performed using the Biotage medium and a high-pressure integrated purification separator (Biotage, Uppsala, Sweden). ^1^H and ^13^C NMR spectra were obtained using a BRUKER AV600 600 MHz spectrometer (Bruker, Billerica, MA, USA) and a JNM-ECA 400 MHz spectrometer (JEOL, Tokyo, Japan), with tetramethylsilane as an internal standard. The multiplicities of the resonance peaks are indicated by the singlet (s), broad singlet (bs), doublet (d), triplet (t), quartet (q), and multiplet (m) peaks. The *J* values are given in hertz, and the relative number of protons was determined by integration. The solvents used for each spectrum are reported. Mass spectra (ESI) were recorded on API3000LC/MS (Applied Biosystems/Sciex, Concord, ON, Canada). The detailed synthetic procedures of titled compounds can be found in the Supplementary materials.

### 3.2. Biological Evaluation

#### 3.2.1. Cloning, Expression, and Purification of the RdRp Domains

The DENV2 RdRp (amino acids 272-900) was cloned into pET15b, with an His6-tag at the *N*-terminus and expressed in *E. coli* BL21pLys cells. Cells were grown at 37 °C to an OD600 of 0.8, induced with 0.4 mM isopropyl β-D-thiogalactopyranoside (IPTG), and further incubated O/N at 15 °C. Cells were harvested by centrifugation, and the pellet was resuspended in 20 mM Na HEPES at pH 7.0, 300 mM NaCl, 5 mM imidazole, DNase I (2 μg/mL), 20 mM MgSO4, and EDTA-free complete protease inhibitors (Roche, Mannheim, Germany). The cells were then lysed using a cell disruptor (Basic Z Bench top 0.75KW; Constant System, Northants, UK). The lysate was clarified via centrifugation at 18,000 rpm for 1 h at 4 °C. The supernatant was purified using a 5 mL bed volume HiTrap nickel-immobilized metal ion affinity chromatography column (G.E. Healthcare, Uppsala, Sweden) connected to an FPLC system (G.E. Healthcare). The column was washed with buffer A (20 mM Na HEPES at pH 7.0, 300 mM NaCl, EDTA-free complete protease inhibitors (Roche)) with 60 mM imidazole. RdRp was then eluted with buffer A + 255 mM imidazole. To remove the *N*-terminal His6-tag, 500 U of thrombin (Sigma, St. Louis, MO, USA) was added to the pooled fraction containing the RdRp, and the mixture was dialyzed overnight at 4 °C against 1 L of buffer A supplemented with 5 mM TCEP. The DENV3 RdRp was purified by size-exclusion chromatography (Superdex 200, Uppsala, Sweden) using the same buffer. The purity of the resulting RdRp was determined using sodium dodecyl sulfate-polyacrylamide gel electrophoresis. The protein was concentrated to 8 mg/mL using a 10-kDa molecular mass cut-off centrifugal concentrator (Millipore, Darmstadt, Germany), divided into aliquots, and stored at −20 °C.

#### 3.2.2. In Vitro DENV Biochemical Assay

RdRp activity was assessed in vitro by synthesizing double-stranded RNA from a single-stranded RNA poly(C) template (Sigma-Aldrich P4903, St. Louis, MO, USA) (1 μg final quantity) and 100 μM GTP. The reaction mixture contained 20 mM Tris/HCl (pH 7.5), 1 mM DTT, 25 mM NaCl, 5 mM MgCl2, 0.3 mM MnCl2, 2 U of RiboLock Ribonuclease inhibitor (Life technologies, Courtaboeuf, France), and 1 μL of PicoGreen Quantitation Reagent (Life technologies). Before initiating the reaction, a final concentration of 1 μM of the protein was added to the mixture, along with the inhibitor (ranging from 0 to 50 μM). The reactions were performed for 1 h at 30 °C using a TECAN Infinite 200 PRO microplate reader, and the increase in PicoGreen fluorescence (Ex/Em = 485/530 nm) was due to the formation of dsRNA. The final fluorescence values were calculated as averages of four independent experiments. Measurements of activity (i.e., linear slope of fluorescence increase in time) vs. inhibitor concentration were used to estimate the IC_50_ values of the two in silico selected compounds using the program GraFit5 (Erithacus software Limited, 2010, Surrey, UK) and the equation Y = (Range/(1 + (X/IC_50_)S)**,** where Range is the value observed for the uninhibited RdRp, and s is a slope factor.

#### 3.2.3. Antiviral Screening and Toxicity Assays

In cell-based antiviral assays, the DENV2 NGC strain (Sino Biological Inc., Beijing, China) and BHK-21 cells (Baby hamster kidney cell line; ATCC CCL10) were used. The viral stock was prepared by inoculating C6/36 cells (*Aedes albopictus* clone C6/36; ATCC CRL166) as described previously [48]. Infected cells were maintained in RPMI 1640 medium (Invitrogen, Burlington, ON, Canada) containing 2% fetal bovine serum (FBS) and 0.5% penicillin–streptomycin stock at 28 °C in a 5% CO_2_ incubator for 7 days, until the cells showed cytopathic effects (CPE). The supernatant was cleared by centrifugation at 2000 *g* for 5 min to remove cell debris and adjusted to 20% FBS. Aliquots of the virus were stored at 80 °C. The 50% tissue culture infective dose (TCID50) was determined using the Promega Viral ToxGlo assay (Fisher Scientific, Ottawa, ON, Canada) in BHK-21 cells, according to the manufacturer’s instructions. Cell-based assays for the evaluation of antiviral activity against DENV were performed as described previously [49,50] with modifications, using the Viral ToxGlo assay (Fisher Scientific, Ottawa, ON, Canada) according to the manufacturer’s instructions. Briefly, BHK-21 cells were plated in 96-well plates at 2500 cells/well in Dulbecco’s modified Eagle’s medium (DMEM) containing 10% FBS and 1% penicillin-streptomycin. Cells were incubated at 37 °C overnight and infected with 100 TCID50 of DENV for 90 min. The medium was replaced with DMEM containing 2.5% FBS and varying concentrations of the test compounds. Cell cultures were maintained at 37 °C in a 5% CO_2_ incubator for 3 days. The Viral Tox reagent was freshly prepared, and 100 μL of the reagent was added to each well. The plates were incubated for at least 10 min and then quantified using a 1450 MicroBeta TriLux microplate scintillation and luminescence counter (PerkinElmer, Waltham, MA, USA). For the off-target cytotoxicity assay, the test compounds were processed as described above, except without DENV infection of the cells. The luminescence readings were plotted against the log transformation of the concentration of the compound. Data were analyzed by nonlinear regression using GraphPad Prism 5.0 software (GraphPad Software, San Diego, CA, USA).

#### 3.2.4. DENV Biochemical Enzyme Assay

The assay incorporated a mixture of components: 100 nM in vitro transcribed DENV 2 mini-genome viral RNA template, which had a length of 380 nucleotides and featured DENV2 linked 5′ to 3′ UTR sequences. Additionally, it contained 20 μM ATP, 20 μM GTP, 20 μM UTP, 5 μM ATTO-CTP, and 100 nM of DENV2 full-length NS5 enzyme. These were all suspended in a solution consisting of 50 mM Tris/HCl at pH 7.5, 10 mM KCl, 1 mM MgCl_2_, 0.3 mM MnCl_2_, 0.001% Triton-X-100, and 10 μM cysteine. Five compounds, with concentrations ranging from 0 to 20 or 100 μM, were serially diluted 2-fold into 384-well white opaque plates (Corning Costar, New York, NY, USA), resulting in a final volume of 5 μL. Subsequently, 5 μL of the enzyme in a 1× assay buffer was added to their respective wells. The plate was incubated at room temperature for 15 min, after which 5 μL of a mixture containing RNA and NTPs was introduced to initiate the reactions. After a reaction time of 120 min, the process was terminated by adding 10 μL of a 2.5× STOP buffer, comprising 200 mM NaCl, 25 mM MgCl_2_, and 1.5 M DEA adjusted to pH 10 using Promega. Additionally, 25 nM of calf intestinal alkaline phosphatase (NEB) was introduced. The mixture was allowed to incubate at room temperature for an additional 60 min. To quantify the results, a Tecan Saffire II microplate reader was employed, with excitation max and emission max wavelengths set at 422 nm and 566 nm, respectively. All data points were analyzed in duplicate wells. Finally, the IC_50_ values were determined using a nonlinear regression curve fit for a sigmoidal dose–response (variable slope) in GraphPad Prism version 3.02 (GraphPad Prism, Inc., San Diego, CA).

### 3.3. Virtual Screening Assay

Molecular docking was performed as described by Schrödinger (Maestro, 2020, Schrödinger Release 2020-1: Schrödinger, LLC, New York, NY, USA). Compounds **27** and **29**, **S-SW-b**, **R-SW-b**, and **SW-d** were saved in *mol2 format.

#### 3.3.1. Protein Preparation

The protein (PDB entry 5K5M) was prepared for docking calculations using the Protein Preparation Workflow (Schrödinger Suite 2020 Protein Preparation Wizard) implemented in the Schrödinger suite and accessible through the Maestro program (Maestro, version 12.3, Schrödinger, 2020). In addition to the default settings, missing side chains and loops were added using Prime. To avoid unnatural interatomic clashes, the ligand–protein complex was refined with restrained minimization performed by Force Field OPLS_2005, setting a maximum RMSD of 0.30 Å. All structures were optimized at neutral pH. Ligand preparation for docking was performed using the LigPrep application (Schrödinger, 2020), which consisted of a series of steps that performed conversions, applied corrections to the structure, generated ionization states and tautomers, and optimized the geometries.

#### 3.3.2. Ligand Preparation

All ligands were designed using Maestro software (Maestro, version 11.5, Schrödinger, 2020). LigPrep (Schrödinger, 2020) was used to generate tautomeric, stereochemical, and ionization variations for all ligands. Finally, partial charges were predicted using the force field OPLS2005.

#### 3.3.3. Docking Simulations

The Ligand docking algorithm was utilized for molecular docking as implemented in the Schrödinger Suite 2020. For calculating the grid box size, the center of the grid box was taken to be the center of the ligand in the crystal structure, and the length of the grid box for the receptor was twice the distance from the ligand center to its farthest ligand atom, plus 10 Å in each dimension. Scoring calculations were performed using extra precision (XP).

#### 3.3.4. Calculation of Binding Energy

The ligand binding free energies were computed using the Prime molecular mechanics-based generalized boron/surface area (MM-GBSA) model of the Schrödinger suite. The MM-GBSA calculations were performed using the variable-dielectric generalized Born solvent model. The minimization was performed with flexibility tolerated for all protein atoms within a 10 Å radius of the ligand.

### 3.4. Molecular Dynamics (MD) Simulation

For molecular dynamics (MD) simulation, Maestro’s Desmond module (Schrödinger, 2020) was used. A system builder panel using the OPLS3 force field was utilized to configure the biological system prior to MD simulation. To solvate the system, an SPC water model was employed, and an orthorhombic box with a 10 Å buffer distance was generated. In this study, MD simulations were conducted for a total duration of 200–500 ns on a GPU, maintaining a temperature of 300 K and a pressure of 1.01325 bar. The Simulation Interaction Diagram shows the ligand–protein interactions, including RMSD images, that determine the stability of the complex. The simulated complex was visualized using PyMOL software (version 2.0, Schrödinger) to observe the polar contacts between the ligand and receptor.

### 3.5. Thermodynamic Calculations: Binding Free Energy Calculation

Calculating the binding free energy can provide a thorough insight of the interaction of the protein–ligand complex. The binding free energy profiles of the DENV NS5 complex with compounds **27**, **29**, **S-SW-b**, **R-SW-b** and **SW-d** were assessed using thermal_mmgbsa.py (Schrödinger, 2020), which employs the Molecular Mechanics Poisson–Boltzmann Surface Area (MMPBSA) approach. A total of 50 snapshots from the final 50 ns of the MD production run were taken into account when estimating the binding free energy for each complex.

## 4. Conclusions

Starting with the potent non-nucleoside inhibitors **27** and **29**, suitable replacement groups for the aryl sulfonamide fragment were identified through virtual compound construction, combined with docking-based virtual screening. Nine compounds were selected for the synthesis. The evaluation included multiple factors, including docking scores, free energy of binding to receptor proteins, predicted ADMET parameters (solubility, permeability, and absorbability), and considerations of structural diversity and feasibility of synthesis. Referring to the relevant literature, we developed a reasonable and feasible route to synthesize these compounds and assessed their anti-dengue virus activity. In the cytopathic effect assay on BHK-21 cells using the DENV2 NGC strain, both compounds **SW-b** and **SW-d** demonstrated comparable or superior activity against DENV2, with IC_50_ values of 3.58 ± 0.29 μM and 23.94 ± 1.00 μM, respectively, compared to that of compound **27** (IC_50_ = 19.67 ± 1.12 μM). Both SW-b and SW-d exhibited low toxicity levels, with CC_50_ values of 24.65 μM and 133.70 μM, respectively, resulting in selectivity indices of 6.89 and 5.58, respectively.

Structural analysis revealed that this increased inhibitory activity could be attributed to the strong π–π interaction between the benzene ring of their S1 fragments and W795, as well as enhancements in hydrogen bonding, hydrophobic interactions, electrostatic interactions, and van der Waals interactions. MD simulations confirmed the stable binding of **SW-b** and **SW-d** to NS5. These findings highlight the promising biological potential of **SW-b** and **SW-d**. However, further studies are required to evaluate their pharmacological and toxicity profiles in in vivo models. Our study provides valuable references and insights for the development of future drugs.

## Figures and Tables

**Table 1:** Structures, docking scores, ADMET parameter, and MM-GBSA ΔG-bind values of the selected target compounds.

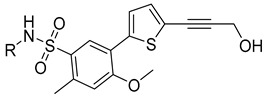						
Entry No.	R	Docking Score	QPPCaco	Aqueous Solubility Level	ADMET Absorption Level	MMGBSA ∆G Bind (kcal/mol)
SW-a	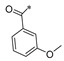	−10.687	108.021	2	0	−89.28
S-SW-b	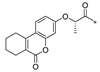	−11.660	84.455	3	2	−115.26
R-SW-b	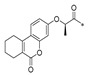	−12.094	115.112	4	1	−108.82
SW-c	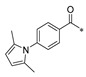	−11.352	114.214	3	1	−98.68
SW-d	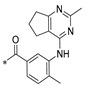	−13.083	138.478	4	2	−98.02
SW-e	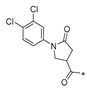	−12.564	123.874	3	0	−98.76
SW-f	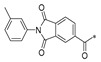	−11.158	90.057	3	1	−99.24
SW-g	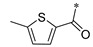	−11.157	130.258	4	2	−87.40
SW-h	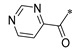	−10.655	187.064	3	0	−81.71
SW-i	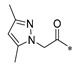	−11.415	150.241	14	2	−93.36
27	-	−12.295	179.421	3	1	−92.13
29	-	−11.995	273.700	3	0	−88.56

**Table 2:** Bioactivity evaluation results of the active compounds.

Entry No.	^a^ DENV-NGC-IC_50_ (μM)	^b^ CC_50_ (μM)	SI (Selectivity Index)
SW-a	57.60 ± 2.37	ND	-
SW-b	3.58 ± 0.29	24.65	6.89
SW-c	41.49 ± 1.37	>200	-
SW-d	23.94 ± 1.00	133.70	5.58
SW-e	>200	69.86	-
SW-f	53.79 ± 4.53	>200	>3.72
SW-g	>200	ND	-
SW-h	>200	ND	-
SW-i	>200	ND	-
BCX4430	41.59 ± 2.95	100	2.40
NITD008	15.45 ± 5.54	20	1.29
27	19.67 ± 1.12	ND	-

^a^ IC_50_ values against DENV-NGC represent the mean ± SD of two individual experiments. ^b^ CC_50_ values; ND, not determined.

**Table 3:** DENV biochemical enzyme evaluation results of the active compounds.

Entry No.	^a^ RdRp-IC_50_ (μM)
SW-b	11.54 ± 1.30
SW-d	13.54 ± 0.32
3′-dATP	30.09 ± 8.26
BCX4430	ND
NITD008	ND

^a^ IC_50_ values against DENV-RdRp Biochemical Enzyme represent the mean ± SD of two individual experiments. ND, not determined.

## Data Availability

Data is contained within the article and supplementary material.

## Supplementary Materials

The following supporting information can be downloaded at: https://www.mdpi.com/article/10.3390/ph16111625/s1.
