# Supplementary material for: Design, Synthesis, Evaluation and Molecular Dynamics Simulation of Dengue Virus NS5-RdRp Inhibitors
Source: Pharmaceuticals (Basel). 2023 Nov 17;16(11):1625. doi: 10.3390/ph16111625 (PMC10674617; doi:10.3390/ph16111625)
Supplement: Supplementary file 1 [file pharmaceuticals-16-01625-s001.zip › pharmaceuticals-2692369-supplementary.pdf]

Supplementary materials

Design, synthesis, biological evaluation and molecular dynamics simulation of Dengue Fever Virus NS5-RdRp inhibitors

Keli Zong [a,b,ξ], Wei Li [b,ξ], Yijie Xu [b ξ], Xu Zhao [c], Ruiyuan Cao [b] *, Hong Yan [a] * and Xingzhou Li

[a]*Faculty of Environment and Life, Beijing University of Technology, 100 Pingleyuan, Beijing, 100124, China*

[b]*Beijing Institute of Pharmacology and Toxicology, 27 Taiping Road, Beijing 100850, China*

[c]*Department of Hepatology, Fifth Medical Center of Chinese PLA General Hospital, 100 West Fourth Ring Road, Beijing100071, China*

[ξ] These authors contribute equally; * Correspondence author.

E-mail addresses: caoruiyuan@bmi.ac.cn (R. Cao), hongyan@bjut.edu.cn (H. Yan), xingzhouli@aliyun.com (X. Li).

Table of contents

The necessary figures in the article......2

The detailed synthetic procedures......5

The ^{1}H NMR and ^{13}C NMR spectra of new compounds......14

The MS spectra of new compounds......23

The HPLC date and method......26

The necessary figures in the article

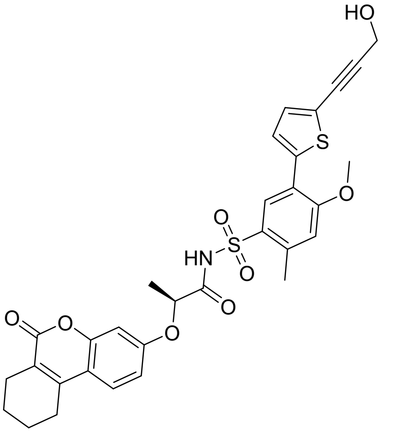

S-SW-b

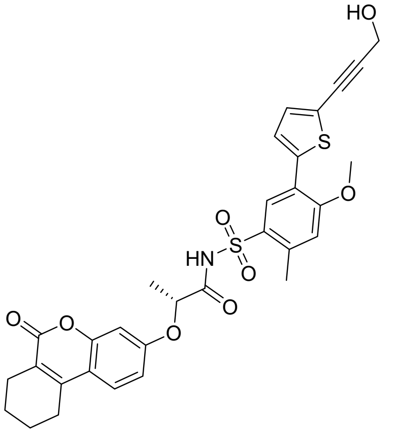

R-SW-b

Figure S1. The structures of S-SW-b and R-SW-b

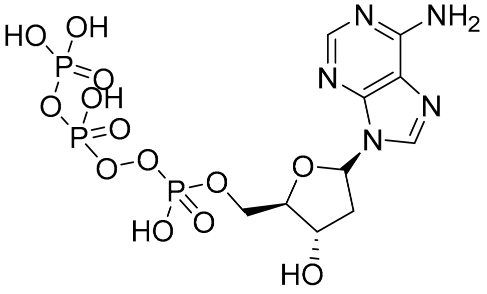

3’-dATP

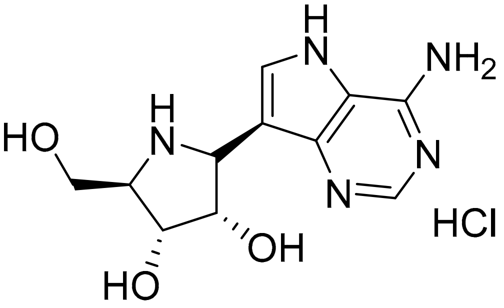

BCX4430

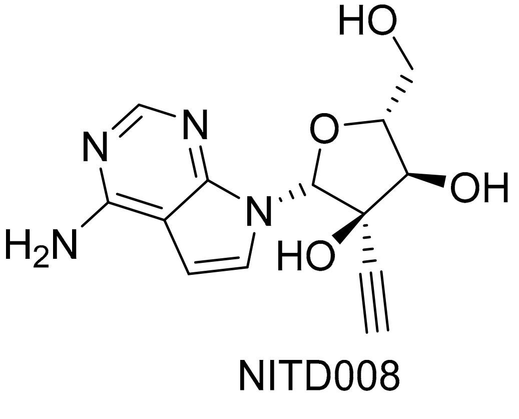

NITD008

Figure S2. Structures of 3’-dATP, BCX4430, and NITD008

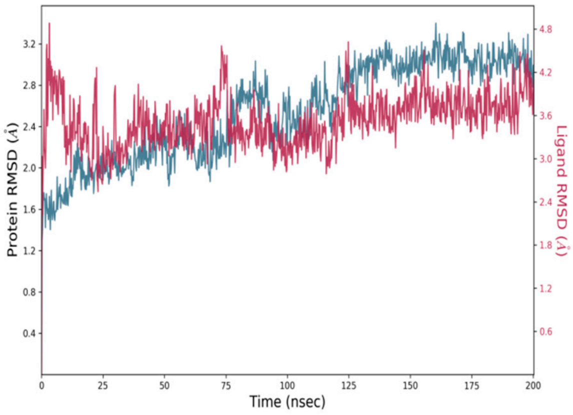

27

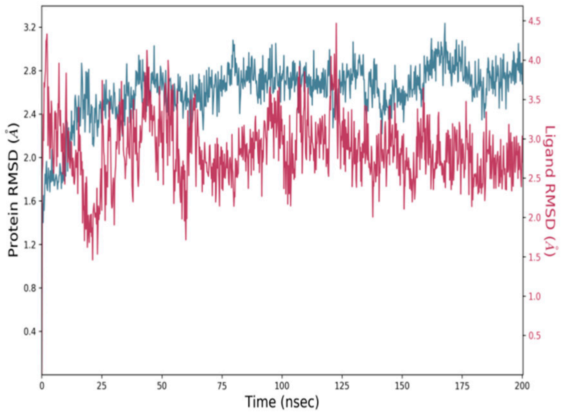

29

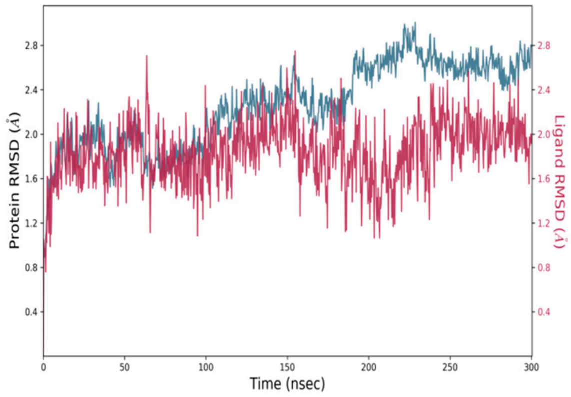

S-SW-b

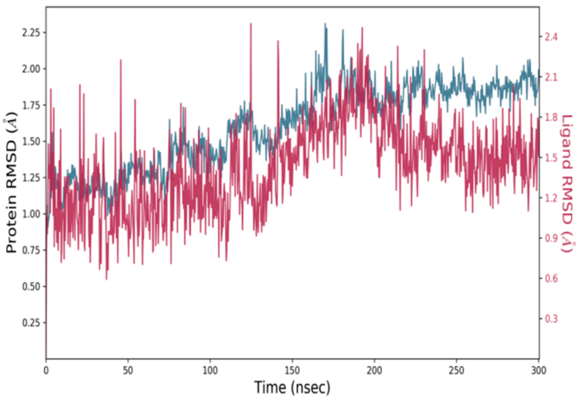

R-SW-b

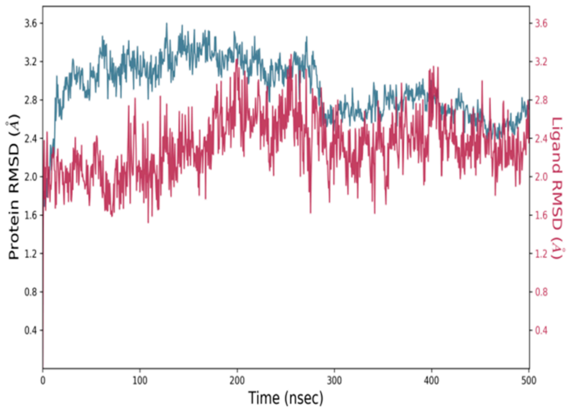

S-SW-d

Figure S3. The RMSD of NS5 protein Cα atoms and Lig fit Prot of Ligand in MD simulations.

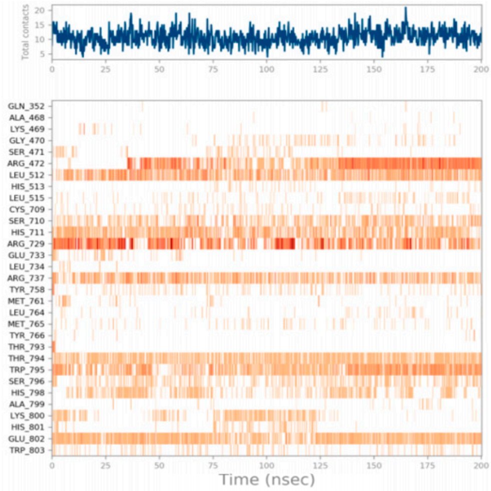

27

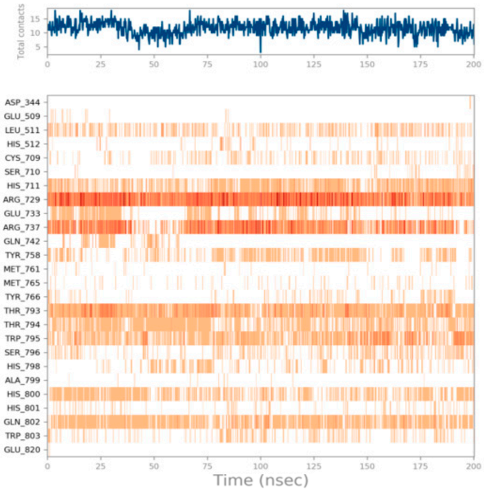

29

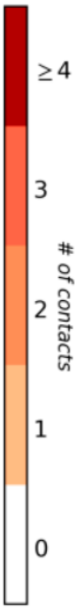

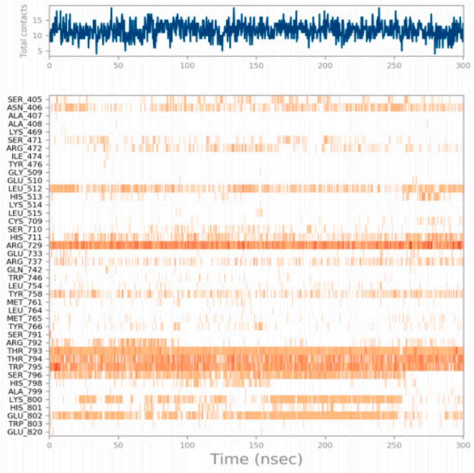

S-SW-b

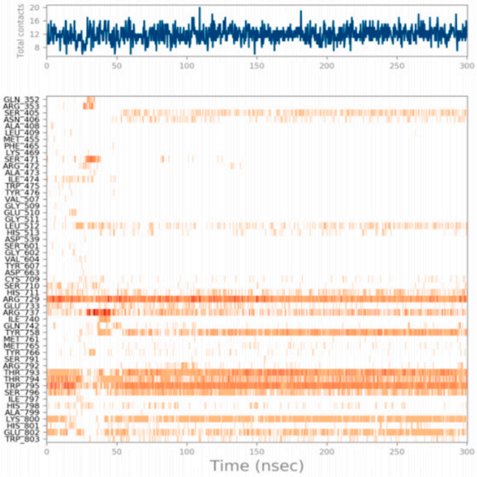

R-SW-b

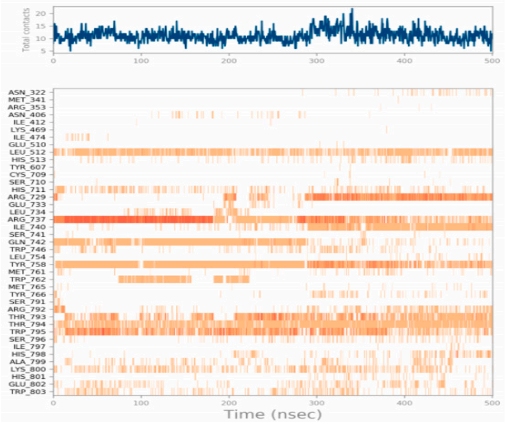

SW-d

Figure S4. Timeline representation of the interactions and contacts (H-bonds, hydrophobic, ionic, water bridges) between compounds.

The detailed synthetic procedures

Synthesis of 9-1-bromo-2-methoxy-4-methylbenzene 2

To a solution of 1-bromo-2-fluoro-4-methylbenzone **1** (20 g, 105.8 mmol) in 1 ml MeOH, 30% MeONa in methanol (47 g, 364.5 mmol) was added. The mixture was stirred at 95 °C for 48 h. The solution was cooled to room temperature, and water (100 ml) was added to quench the reaction. The resulting solution was extracted with CH_2Cl_2 (3×50 ml), and the organic layer was washed with brine (3×100 ml), dried over Na_2SO_4, filtered, and evaporated. The residue was purified by flash chromatography (silica gel, petroleum ether/ethyl acetate) to yield compound **2** as a colorless solid (9.5 g, 50%). ^{1}H NMR (400MHz, Chloroform-*d*) δ 7.41 (s, 1H), 6.72 (m, 2H), 3.88 (s, 3H), 2.33 (s, 3H).

5-bromo-4-methoxy-2-methylbenzenesulfonchloride 3

Chlorosulfonic acid (5.8 g, 49.6 mmol) was slowly added to anhydrous CH_2Cl_2 (25 ml) containing 1-bromo-2-methoxy-4-methylbenzene **2** (5 g, 24.8 mmol). The mixture was stirred at 80°C for 2 h. The solution was cooled to room temperature, and 0°C water was added to quench the reaction. The resulting solution was extracted with CH_2Cl_2 (3×15 ml), and the organic layer was washed with brine (3×50 ml), dried over Na_2SO_4, filtered, and evaporated to obtain compound **3**, which was used directly in the subsequent step without further purification.

5-bromo-4-methoxy-2-methylbenzenesulfonamide 4

A mixture of compound **3** in MeOH (5 ml) was added to $NH_3 \cdot H_2O$ (30 ml) at 0 °C and stirred at room temperature for 12 h. The turbid solution was filtered to obtain a white solid, which was evaporated under vacuum to obtain compound **4** as a white solid (2.9 g, 40%). ^{1}H NMR (400 MHz, DMSO-d_6) δ 7.91 (s, 1H), 7.37 (s, 2H), 7.10 (s, 1H), 3.88 (s, 3H), 2.50 (s, 3H). ESI-MS: m/z 281.95 $[M+H]^+$.

Tert-butyl ((5-bromo-4-methoxy-2-methylphenyl)sulfonyl)carbamate 5

The mixed solution of 5-bromo-4-methoxy-2-methylbenzenesulfonamide **4** (4.7 g, 16.8 mmol) and $(Boc)_2O$ (5.5 g, 25.2 mmol) in CH_2Cl_2 (90 ml) was added dropwise to TEA (4.7 ml, 33.6 mmol) at room temperature. After stirring overnight, the solution was diluted with CH_2Cl_2 (30 ml); washed with HCl (2×20 ml, 1 M), H_2O (2×20 ml) and brine (2×20 ml); dried over Na_2SO_4; filtered; and evaporated. The residue was purified using flash chromatography (silica gel and petroleum ether/ethyl acetate) to

obtain compound **5** as a white solid (4.61 g, 72.2%). ^{1}H NMR (400 MHz, Chloroform-*d*) δ 8.24 (s, 1H), 7.75 (s, 1H), 6.78 (s, 1H), 3.96 (s, 3H), 2.61 (s, 3H), 1.37 (s, 9H). ESI-MS: m/z 381.96 $[M+H]^+$.

Tert-butyl ((4-methoxy-2-methyl-5-(thiophen-2-yl)phenyl)sulfonyl)carbamate 6

To a solution of tert-butyl ((5-bromo-4-methoxy-2-methylphenyl)sulfonyl)carbamate **5** (2 g, 5.26 mmol) in 1,2-Dimethoxyethane/H_2O (3:1, 40 ml), thiophen-2-ylboronic acid (1.34 g, 10.52 mmol), Na_2CO_3 (1.4 g, 13.15 mmol), and Bis(triphenylphosphine)palladium(II) chloride were successively added at 80°C. The reaction mixture was stirred under N_2 atmosphere for 2 h. The solution was then cooled to room temperature and diluted with ethyl acetate (70 ml). The reaction mixture was washed with brine (3×20 ml), dried over Na_2SO_4, filtered, and evaporated. The organic layer was dried over Na_2SO_4, filtered, and evaporated under reduced pressure to obtain intermediate **6**. The residue was purified using flash chromatography (silica gel and petroleum ether/ethyl acetate) to obtain compound **6** (810 mg, 45%). ^{1}H NMR (400 MHz, Chloroform-*d*) δ 8.36 (s, 1H), 7.54 (s, 1H), 7.38 – 7.32 (m, 2H), 7.10 (s, 1H), 6.87 (s, 1H), 4.00 (s, 3H), 2.67 (s, 3H), 1.33 (s, 9H).

tert-butyl ((5-(5-bromothiophen-2-yl)-4-methoxy-2-methylphenyl)sulfonyl)carbama--te 7

To a solution of tert-butyl ((4-methoxy-2-methyl-5-(thiophen-2-yl)phenyl) sulfonyl)carbamate **6** (960 mg, 2.5 mmol) in DMF, NBS (445 mg, 2.5 mmol) and BPO were added at 80°C. The mixture was stirred at 90°C for 2 h, then cooled to room temperature before adding 0°C water (100 ml). The solid precipitated overnight. The precipitated solid was collected by filtration and dried under vacuum to yield compound **7** as a light yellow solid (718 mg, 74.5%). ^{1}H NMR (400 MHz, Methanol-d_4) δ 8.23 (s, 1H), 7.35 (s, 1H), 7.09 (s, 2H), 3.62 (s, 3H), 2.69 (s, 3H), 1.18 (s, 9H).

5-(5-bromothiophen-2-yl)-4-methoxy-2-methylbenzenesulfonamid 8

To a solution of tert-butyl ((5-(5-bromothiophen-2-yl)-4-methoxy-2-methylphenyl)sulfonyl)carbamate **7** (5 g,7.2 mmol) in CH_2Cl_2 (15 ml), TFA (15 ml) was added dropwise and stirred for 6 h. Then, it was diluted with CH_2Cl_2 (90 ml) and washed with brine (3×30 ml). The combined organic extracts were dried over Na_2SO_4, filtered, and concentrated to obtain compound **8** (6.38 g). ^{1}H NMR (400 MHz,

Methanol-d_4) δ 8.23 (s, 2H), 7.33 (s, 1H), 7.15 – 7.04 (m, 2H), 6.99 (d, J = 3.9 Hz, 1H), 4.01 (s, 3H), 2.68 (s, 3H). ESI-MS: m/z 361.93 $[M+H]^+$.

4-methoxy-2-methyl-5-(5-(3-((tetrahydro-2H-pyran-2-yl)oxy)prop-1-yn-1-yl)thiophen-2-yl)benzenesulfonamide 9

2-prop-2-ynoxyoxane (664 mg, 4.74 mmol), CuI (30 mg, 0.316 mmol), TEA (1.6 g, 15.8 mmol) and $Pd(dppf)Cl_2·CH_2Cl_2$ (194 mg, 0.237 mmol) were added to the 1,4-dioxane (45 ml) solution of 5-(5-bromothiophen-2-yl)-4-methoxy-2-methylbenzenesulfonamide **8** (570 mg, 1.58 mmol) under dark stirring at 86°C in an N_2 atmosphere for 12 hours. The reaction mixture was then cooled to room temperature. The reaction mixture was diluted with ethyl acetate (45 ml), and the solution was washed with brine (3×40 ml). The combined organic extracts were dried over Na_2SO_4, filtered, and concentrated under vacuum. The residue was purified using flash chromatography (silica gel and petroleum ether/ethyl acetate) to obtain compound **9** (370 mg, 56%). ^1H NMR (400 MHz, Chloroform-d) δ 8.26 (s, 2H), 7.35 (s, 1H), 7.26 (m, 2H), 7.19 (s, 1H), 4.81 (s, 1H), 4.52 (d, J = 9.9 Hz, 2H), 4.01 – 3.85 (m, 5H), 2.69 (s, 3H), 1.67 – 1.51 (m, 6H). ESI-MS: m/z 422.09 $[M+H]^+$.

Synthesis of 2-((6-oxo-7,8,9,10-tetrahydro-6H-benzo[c]chromen-3-yl)oxy)propanoic acid 25b

Resorcinol **10** (2.5 g, 23 mmol) and Ethyl 2-oxocyclohexanecarboxylate **11** (4.7 g, 27.6 mmol) were placed in a reaction vial. To this mixture, $MeSO_3H$ (2.2 g, 23 mmol) and Acidic Al_2O_3 (0.2 g) were added. The reaction mixture was stirred at room temperature for 30 min. The resulting solution was then mixed with anhydrous EtOH (230 ml) and stirred at 70°C for 1 h. Afterward, it was cooled to room temperature before adding 0°C water, resulting in the separation of solid material. The precipitate was collected by filtration and dried under vacuum to yield compound **12** as a white solid (2 g, 77% yield). This compound was used directly in the subsequent steps without further purification.

To a solution of 3-hydroxy-7,8,9,10-tetrahydro-6H-benzo[c]chromen-6-one **12** (1 g, 4.6 mmol) in DMF (20 ml), ethyl 2-bromopropanoate (2.5 g, 13.8 mmol) and K_2CO_3 (1.9 g, 13.8 mmol) were added. The reaction mixture was stirred at 80°C for 1 h, then cooled to room temperature before being quenched with 0°C water. The mixture was

then extracted with ethyl acetate (3×40 ml). The organic solvent was evaporated under reduced pressure to yield a yellowish ester oil. The residue was stirred overnight in 10% NaOH solution. The solution was acidified with aq 2 M HCl until reaching a pH of 2, resulting in the gradual formation of precipitate. The precipitated solid was collected by filtration and dried under vacuum to obtain compound **25b** as a yellow solid (1.2 g, 80%). ^{1}H NMR (400 MHz, Chloroform-*d*) δ 8.87 (s, 1H), 7.31 (d, *J* = 8.8 Hz, 1H), 6.76 (d, *J* = 6.2 Hz, 1H), 6.63 (s, 1H), 4.75 (q, *J* = 6.8 Hz, 1H), 2.58 (d, *J* = 6.4 Hz, 2H), 2.41 (t, *J* = 6.0 Hz, 2H), 1.87 – 1.51 (m, 7H). ESI-MS: m/z 289.10 $[M+H]^+$.

Synthesis of 4-(2,5-dimethyl-1H-pyrrol-1-yl)benzoic acid 25c

To a solution of 4-aminobenzoic acid **14** (1.37 g, 10 mmol) in AcOH (8 ml) was added 2,5-hexanedione **13** (1.37 g, 12 mmol), and the mixture was stirred at 95°C for 30 min, then cooled to room temperature before adding 0°C water, and the red solid was separated. The precipitated solid was collected by filtration and dried under vacuum to obtain compound **25c** as a red solid (1.9 g, 71%). ^{1}H NMR (400 MHz, DMSO-d_6) δ 13.10 (s, 1H), 8.03 (m, 2H), 7.36 (m, 2H), 5.79 (m, 2H), 1.95 (s, 6H). ESI-MS: m/z 216.10 $[M+H]^+$.

Synthesis of 4-methyl-3-((2-methyl-6,7-dihydro-5H-cyclopenta[d]pyrimidin-4-yl)amino)benzoic acid 25d

A mixture of methyl 3-oxocyclopentane-1-carboxylate **15** (1.25 ml, 9.9 mmol), ethylamine hydrochloride **16** (1.42 g, 15.6 mmol), and *t*-BuOK (3.1 g, 27.7 mmol) in tert-butanol was stirred and the reaction mixture was refluxed overnight. The solution was cooled to room temperature and filtered through methanol. The resulting filtrate was evaporated to obtain compound **17**, which was used directly in subsequent steps without further purification.

A mixture of **17** and $POCl_3$ was refluxed for 3 h, after which it was cooled to room temperature and concentrated. The resulting residue was diluted with CH_2Cl_2 (50 ml) and neutralized with ammonia in an ice bath. The organic material was washed with water (3×30 ml), dried over Na_2SO_4, filtered, and evaporated to obtain **18**, which was used directly in the subsequent steps without further purification.

To a solution of methyl 3-amino-4-methylbenzoate (10.0 g, 66.0 mmol) and IPA (200 ml), compound **18** (13.0 g, 80.0 mmol) and HCl (2 ml) were added. The mixture

was stirred at 95°C for 3 h, then cooled to 2–8°C overnight, resulting in the separation of the solid. The precipitated solid was collected via filtration, washed with cold IPA (2×50 ml), and dried under vacuum to obtain an intermediate. A THF (7 ml)–H_2O (7 ml) solution containing lithium hydroxide monohydrate (100 mg, 2 mmol) in THF (7 mL)–H_2O (7 mL) was slowly added to the intermediate (200 mg, 0.67 mmol). After stirring at room temperature for 4 h, the solution was acidified with aq 2M HCl to reach pH 2, resulting in the gradual formation of a precipitate. The precipitated solid was collected by filtration and dried under vacuum to obtain compound **25d** as a white solid (100 mg, 58% yield). ^{1}H NMR (400 MHz, Methanol-*d_4*) δ 7.89 (m, 2H), 7.44 (s, 1H), 3.29 (m, 2H), 3.02 (d, *J* = 8.8 Hz, 4H), 2.47 (s, 3H), 2.29 (s, 3H). ESI-MS: m/z 284.13 $[M+H]^+$.

Synthesis of 1-(3,4-dichlorophenyl)-5-oxopyrrolidine-3-carboxylic acid 25e

A mixture of 3,4-dichloroaniline **19** (1.62 g, 10 mmol) and itaconic acid **20** (1.3 g, 10 mmol) was stirred at 100°C and monitored by TLC until the end of the reaction. After the mixture was cooled to 0°C, dissolved in saturated $NaHCO_3$ solution, and filtered to remove insoluble solids, the solution was acidified with aq 2M HCl until pH=2, and precipitate gradually appeared. The precipitated solid was collected by filtration and dried under vacuum to obtain compound **25e** as a white solid (800 mg, 89%). ^{1}H NMR (400 MHz, DMSO-*d_6*) δ 7.98 (d, *J* = 1.8 Hz, 1H), 7.58 (d, *J* = 1.8 Hz, 2H), 4.09 – 3.87 (m, 2H), 3.31 (d, *J* = 2.8 Hz, 1H), 2.72 (qd, *J* = 8.2, 6.4 Hz, 2H). ESI-MS: m/z 274.00 $[M+H]^+$.

Synthesis of 1,3-dioxo-2-(m-tolyl)isoindoline-5-carboxylic acid 25f

A mixture of trimellitic anhydride **21** (500 mg, 2.6 mmol) and *m*-toluidine **22** (279 mg, 2.6 mmol) in AcOH (10 ml) was stirred at 130°C for 16 h. The solution was cooled to room temperature, and a yellow precipitate appeared. The precipitated solid was collected by filtration and dried under vacuum to obtain compound **25f** as a yellow solid (520 mg, 70%). ^{1}H NMR (400 MHz, DMSO-*d_6*) δ 8.43 (d, *J* = 2.8 Hz, 1H), 8.30 (s, 1H), 8.09 (d, *J* = 12.8 Hz, 1H), 7.42 (s, 1H), 7.29 (m, 3H), 2.37 (s, 3H). ESI-MS: m/z 280.06 $[M+H]^+$.

Synthesis of 2-(3,5-dimethyl-1H-pyrazol-1-yl)acetic acid 25i

A mixture of 3,5-dimethyl-1*H*-pyrazole **23** (1g, 10.4 mmol), ethyl 2-bromoacetate

24 (1.9 g, 11.4 mmol) and K_2CO_3 (4.3 g, 31.2 mmol) in acetone (10 ml) was stirred, and the reaction mixture was refluxed overnight. The solution was cooled to room temperature, filtered to remove insoluble matter, and concentrated in vacuum. The residue was extracted with ether (20 ml) and water (2×20 ml), and the combined organic extracts were dried over Na_2SO_4, filtered, and concentrated to obtain an oily residue. The oily residue was dissolved in n-hexane at 50°C, and insoluble impurities were removed from the hot solution via filtration. The filtrate was then cooled at 0°C, resulting in the formation of a solid. The solid was collected via filtration and subsequently dried under vacuum to obtain an intermediate product. To a solution of the intermediate in water (7 ml), NaOH (876 mg, 21.9 mmol) was added at 0°C. The reaction was stirred at room temperature until TLC monitoring was completed, and then the solution was acidified with aq 1M HCl to reach a pH of 3. The acidified aqueous layer was extracted using CH_2Cl_2 (3×50 ml). The combined organic extracts were dried over Na_2SO_4, filtered, and concentrated to obtain compound **25i** as a white crystal (1.95 g, 80%). ^{1}H NMR (400 MHz, DMSO-d_6) δ 5.76 (s, 1H), 4.70 (s, 2H), 2.08 (s, 3H), 2.02 (s, 3H). ESI-MS: m/z 155.08 $[M+H]^+$.

Synthesis of SW-(a-i)

The target product, **SW-(a-i)**, was prepared from key intermediates **25c-i** and **9** according to the procedure described for the synthesis of **SW-c**.

Synthesis of N-((5-(5-(3-hydroxyprop-1-yn-1-yl)thiophen-2-yl)-4-methoxy-2-methylphenyl)sulfonyl)-3-methoxybenzamide SW-a

To a solution of **9** (160 mg, 0.38 mmol) in CH_2Cl_2 (10 ml), carboxylic acid **25a** (87 mg, 0.57 mmol), EDCI (46 mg, 0.76 mmol) and DMAP (139 mg, 1.14 mmol) were added at 35°C, and the mixture was stirred for 12 h. The reaction mixture was diluted with CH_2Cl_2 (30 ml). The solution was washed with saturated brine (3×20 ml) and dried over Na_2SO_4, filtered, and concentrated to obtain an intermediate (54 mg, 45%).

To a solution of the intermediate (120 mg, 0.22 mmol) in MeOH (5 ml), saturated NH_4Cl solution (2 ml) and 0.5% citric acid solution (1 ml) were added at 45°C, and the mixture was stirred overnight. The reaction mixture was concentrated and diluted with CH_2Cl_2 (30 ml). The solution was washed with saturated brine (3×10 ml) and dried over Na_2SO_4, filtered, and concentrated under a vacuum. The residue was purified using

flash chromatography (silica gel and petroleum ether/ethyl acetate) to obtain the target compound **SW-a** (60 mg, 48%). 99% HPLC purity (254 nm). m.p. 162℃. ^{1}H NMR (400 MHz, DMSO-d_6) δ 12.68 (s, 1H), 8.30 (s, 1H), 7.52 (d, J = 3.8 Hz, 1H), 7.48 – 7.35 (m, 3H), 7.31 (d, J = 3.8 Hz, 1H), 7.23 – 7.16 (m, 2H), 4.34 (s, 2H), 4.00 (s, 3H), 3.79 (s, 3H), 2.64 (s, 3H). ^{13}C NMR (101 MHz, DMSO-d_6) δ 163.64 , 160.40 , 158.76 , 158.61 , 155.77 , 140.01 , 138.29 , 132.64 , 130.64 , 129.79 , 126.20 , 119.98 , 119.63 , 116.24 , 95.70 , 77.48 , 56.99 , 50.12 , 20.45 . HRMS (ESI), m/z 472.0886 [M+H]$^+$, calculated for $C_{23}H_{22}NO_6S_2$, 472.0889.

Synthesis of N-((5-(5-(3-hydroxyprop-1-yn-1-yl)thiophen-2-yl)-4-methoxy-2-methylphenyl)sulfonyl)-2-((6-oxo-7,8,9,10-tetrahydro-6H-benzo[c]chromen-3-yl)oxy)propanamide SW-b

To a solution of **9** in CH_2Cl_2 (20 ml), carboxylic acid **25b** (277 mg, 0.96 mmol), HATU (366 mg, 0.96 mmol), and DIPEA (186 mg, 1.14 mmol) were added at 35°C, and the mixture was stirred for 12 h. The post-processing operation of the resulting solution can be referenced from **SW-a**. The yield of **SW-b** was 45%. 97% HPLC purity (254 nm). m.p. 170°C. ^{1}H NMR (600 MHz, DMSO-d_6) δ 8.13 (s, 1H), 7.51 (d, J = 8.6 Hz, 1H), 7.38 (d, J = 3.9 Hz, 1H), 7.24 (d, J = 3.9 Hz, 1H), 7.11 (s, 1H), 6.77 (d, J = 8.8 Hz, 1H), 6.60 (s, 1H), 4.95 (s, 1H), 4.33 (s, 2H), 3.95 (s, 3H), 2.70 (s, 2H), 2.59 (s, 3H), 2.39 (d, J = 4.6 Hz, 2H), 1.80 – 1.68 (m, 4H), 1.46 (d, J = 6.7 Hz, 3H). ^{13}C NMR (101 MHz, DMSO-d_6) δ 167.33 , 167.23 , 138.77 , 134.78 , 132.55 , 132.31 , 131.92 , 129.23 , 129.13 , 128.29 , 125.36 , 124.97 , 123.54 , 123.15 , 115.24 , 95.29 , 77.68 , 56.55 , 50.11 , 21.34 , 20.70 . HRMS (ESI), m/z 608.1407 [M+H]$^+$, calculated for $C_{31}H_{30}NO_8S_2$, 608.1413 and m/z 630.1239 [M+Na]$^+$ (100%), calculated for $C_{31}H_{30}NNaO_8S_2$, 630.1232.

Synthesis of 4-(2,5-dimethyl-1H-pyrrol-1-yl)-N-((5-(5-(3-hydroxyprop-1-yn-1-yl)thiophen-2-yl)-4-methoxy-2-methylphenyl)sulfonyl)benzamide SW-c

To a solution of the carboxylic acid **25c** in CH_2Cl_2 was added EDCI (100 mg, 0.52 mmol), HOBT (71 mg, 0.52 mmol), and **9** (110 mg, 0.26 mmol) dissolved in CH_2Cl_2, and then DIPEA (100 mg, 0.78 mmol) was added by drop at 35°C, and the mixture was stirred for 12 h. For the post-processing operation of the resulting solution, refer to the **SW-a.** The yield of **SW-c** was 65%. 96% HPLC purity (254 nm). m.p. 240°C. ^{1}H NMR

(400 MHz, Methanol-d_4) δ 8.47 (s, 1H), 7.96 (d, J = 8.6 Hz, 2H), 7.52 (d, J = 4.0 Hz, 1H), 7.34 (d, J = 8.6 Hz, 2H), 7.21 (d, J = 3.9 Hz, 1H), 7.11 (s, 1H), 5.83 (s, 2H), 4.43 (s, 2H), 4.03 (s, 3H), 2.73 (s, 3H), 2.00 (s, 3H), 1.29 (s, 3H). ^{13}C NMR (101 MHz, DMSO-d_6) δ 158.14 , 142.64 , 139.39 , 138.60 , 132.57 , 130.44 , 129.92 , 128.35 , 128.01 , 125.99 , 119.84 , 119.39 , 115.99 , 107.12 , 95.57 , 77.47 , 65.39 , 56.85 , 50.09 , 20.44 , 15.64 , 13.36 . HRMS (ESI), m/z 535.1356 [M+H]$^+$, calculated for $C_{28}H_{27}N_2O_5S_2$, 535.1361.

Synthesis of 4-(2,5-dimethyl-1H-pyrrol-1-yl)-N-((5-(5-(3-hydroxyprop-1-yn-1-yl)thiophen-2-yl)-4-methoxy-2-methylphenyl)sulfonyl)benzamide **SW-d**

The target compound **SW-d** was synthesized from **9** and carboxylic acid **25d** using methods similar to those described for the preparation of **SW-c**. The yield of **SW-d** was 55%. 99% HPLC purity (254 nm). m.p. 168°C. ^{1}H NMR (400 MHz, DMSO-d_6) δ 8.23 (s, 1H), 7.79 (s, 1H), 7.73 (d, J = 6.4 Hz, 1H), 7.46 (d, J = 4.0 Hz, 1H), 7.33 (d, J = 8.1 Hz, 1H), 7.29 (d, J = 3.9 Hz, 1H), 7.08 (s, 1H), 4.33 (s, 2H), 3.96 (s, 3H), 2.91 (t, J = 7.5 Hz, 2H), 2.74 – 2.65 (m, 2H), 2.59 (s, 3H), 2.37 (s, 3H), 2.18 (s, 3H), 2.11 – 2.03 (m, 2H). ^{13}C NMR (101 MHz, DMSO-d_6) δ 167.32 , 167.21 , 138.76 , 138.46 , 134.77 , 132.53 , 132.30 , 131.90 , 129.22 , 129.12 , 128.28 , 125.35 , 124.96 , 123.53 , 123.14 , 122.06 , 115.23 , 95.28 , 77.67 , 56.54 , 50.11 , 21.33 , 20.70 . HRMS (ESI), m/z 603.1730 [M+H]$^+$, calculated for $C_{31}H_{31}N_4O_5S_2$, 603.1736.

Synthesis of 1-(3,4-dichlorophenyl)-N-((5-(5-(3-hydroxyprop-1-yn-1-yl)thiophen-2-yl)-4-methoxy-2-methylphenyl)sulfonyl)-5-oxopyrrolidine-3-carboxamide SW-e

The target compound **SW-e** was synthesized from **9** and carboxylic acid **25e** using methods similar to those described for the preparation of **SW-c**. The yield of **SW-e** was 56%. 96% HPLC purity (254 nm). m.p. 168°C. ^{1}H NMR (400 MHz, DMSO-d_6) δ 8.14 (s, 1H), 7.99 (d, J = 2.4 Hz, 1H), 7.60 (d, J = 8.9 Hz, 1H), 7.55 (dd, J = 8.9, 2.5 Hz, 1H), 7.43 (d, J = 4.0 Hz, 1H), 7.27 (d, J = 3.9 Hz, 1H), 7.09 (s, 1H), 5.42 (s, 1H), 4.33 (d, J = 5.0 Hz, 2H), 4.13 (s, 2H), 3.96 (s, 3H), 3.83 (dd, J = 9.8, 5.3 Hz, 1H), 2.72 (dd, J = 17.2, 9.5 Hz, 1H), 2.58 (d, J = 8.4 Hz, 4H). ^{13}C NMR (101 MHz, DMSO-d_6) δ 172.68 , 158.01 , 139.42 , 139.21 , 138.63 , 132.55 , 131.56 , 130.99 , 130.05 , 126.15 , 125.88 , 122.67 , 121.10 , 119.60 , 119.27 , 115.91 , 95.53 , 77.47 , 56.81 , 50.09 , 37.06 , 35.65 , 20.32 . HRMS (ESI), m/z 593.0370 [M+H]$^+$, calculated for $C_{26}H_{23}Cl_2N_2O_6S_2$,

593.0375 and m/z 615.3480 [M+Na]$^+$ (100%), calculated for $C_{26}H_{22}Cl_2N_2NaO_6S_2$, 615.3494.

Synthesis of N-((5-(5-(3-hydroxyprop-1-yn-1-yl)thiophen-2-yl)-4-methoxy-2-methylphenyl)sulfonyl)-1,3-dioxo-2-(m-tolyl)isoindoline-5-carboxamide SW-f

The target compound **SW-f** was synthesized from **9** and carboxylic acid **25f** using methods similar to those described for the preparation of **SW-c**. The yield of **SW-f** was 55%. 98% HPLC purity (254 nm). m.p. 194°C. ^{1}H NMR (400 MHz, DMSO-d_6) δ 8.30 (d, J = 8.8 Hz, 2H), 8.15 (s, 1H), 7.86 (d, J = 7.5 Hz, 1H), 7.45 – 7.29 (m, 3H), 7.22 (dd, J = 15.9, 4.4 Hz, 3H), 6.93 (s, 1H), 5.37 (s, 1H), 4.30 (s, 2H), 3.88 (s, 3H), 2.52 (s, 3H), 2.31 (s, 3H). ^{13}C NMR (101 MHz, DMSO-d_6) δ 167.33 , 167.23 , 138.77 , 138.47 , 134.78 , 132.55 , 132.31 , 131.92 , 129.23 , 129.13 , 128.29 , 125.36 , 124.97 , 123.54 , 123.15 , 122.07 , 115.24 , 95.29 , 77.68 , 56.55 , 50.11 , 40.27 , 21.34 , 20.70 . HRMS (ESI): m/z 601.1101 [M+H]$^+$, calculated for $C_{31}H_{25}N_2O_7S_2$; 601.1103 and m/z 623.0916 [M+Na]$^+$ (100%), calculated for $C_{31}H_{25}N_2NaO_7S_2$; 623.0923.

Synthesis of N-((5-(5-(3-hydroxyprop-1-yn-1-yl)thiophen-2-yl)-4-methoxy-2-methylphenyl)sulfonyl)-5-methylthiophene-2-carboxamide SW-g

The target compound **SW-g** was synthesized from **9** and carboxylic acid **26g** using methods similar to those described for the preparation of **SW-c**. The yield of **SW-g** was 58%. 95% HPLC purity (254 nm). m.p. 202°C. ^{1}H NMR (400 MHz, DMSO-d_6) δ 8.12 (d, J = 4.5 Hz, 2H), 7.39 (d, J = 3.9 Hz, 1H), 7.24 (d, J = 3.9 Hz, 1H), 6.97 (s, 1H), 6.68 (s, 1H), 5.38 (s, 1H), 4.30 (s, 2H), 3.90 (s, 3H), 2.53 (s, 3H), 2.36 (s, 3H). ^{13}C NMR (101 MHz, DMSO-d_6) δ 163.64 , 160.40 , 158.74 , 158.61 , 155.77 , 140.01 , 138.29 , 132.64 , 130.66 , 129.80 , 126.20 , 122.99 , 119.98 , 119.64 , 116.24 , 116.24 , 95.70 , 77.48 , 56.99 , 50.12 , 49.11 , 20.45 . HRMS (ESI), m/z 462.0496 [M+H]$^+$, calculated for $C_{21}H_{20}NO_5S_3$, 462.0504.

Synthesis of N-((5-(5-(3-hydroxyprop-1-yn-1-yl)thiophen-2-yl)-4-methoxy-2-methylphenyl)sulfonyl)pyrimidine-4-carboxamide SW-h

The target compound **SW-h** was synthesized from **9** and carboxylic acid **26h** using methods similar to those described for the preparation of **SW-c**. The yield of **SW-h** was 50%. 97% HPLC purity (254 nm). m.p. 182°C. ^{1}H NMR (400 MHz, DMSO-d_6) δ 9.36 (s, 1H), 9.06 – 8.97 (m, 1H), 8.29 (s, 1H), 7.91 – 7.82 (m, 1H), 7.48 (d, J = 3.6 Hz, 1H),

7.27 (d, $J = 3.7$ Hz, 1H), 7.16 (s, 1H), 4.30 (s, 2H), 3.96 (s, 3H), 2.61 (s, 3H). ^{13}C NMR (101 MHz, DMSO-d_6) δ 163.64 , 160.40 , 158.63 , 155.77 , 140.01 , 138.29 , 132.64 , 130.66 , 129.80 , 126.20 , 122.99 , 119.98 , 119.64 , 116.24 , 95.70 , 77.48 , 56.99 , 50.12 , 20.45 . HRMS (ESI), m/z 444.0681 $[M+H]^+$, calculated for $C_{20}H_{18}N_3O_5S_2$, 444.0688 and m/z 466.0478 $[M+Na]^+$ (100%), calculated for $C_{20}H_{17}N_3NaO_5S_2$, 466.0507.

Synthesis of 2-(3,5-dimethyl-1H-pyrazol-1-yl)-N-((5-(5-(3-hydroxyprop-1-yn-1-yl)thiophen-2-yl)-4-methoxy-2-methylphenyl)sulfonyl)acetamide SW-i

The target compound **SW-i** was synthesized from **9** and carboxylic acid **26i** using methods similar to those described for the preparation of **SW-c**. The yield of **SW-i** was 40%. 98% HPLC purity (254 nm). m.p. 172°C.^{1}H NMR (400 MHz, DMSO-d_6) δ 8.09 (s, 1H), 7.40 (d, $J = 4.0$ Hz, 1H), 7.26 (d, $J = 3.9$ Hz, 1H), 7.04 (s, 1H), 5.70 (s, 1H), 5.41 (s, 1H), 4.49 (s, 2H), 4.33 (s, 2H), 3.95 (s, 3H), 2.57 (s, 3H), 2.01 (d, $J = 5.1$ Hz, 6H). ^{13}C NMR (101 MHz, DMSO-d_6) δ 167.00 , 158.56 , 146.97 , 140.57 , 139.57 , 138.24 , 132.59 , 130.24 , 126.15 , 122.94 , 119.61 , 116.27 , 105.51 , 95.67 , 77.47 , 56.99 , 51.52 , 50.12 , 20.30 , 13.68 , 10.91 . HRMS (ESI), m/z 474.1152 $[M+H]^+$, calculated for $C_{22}H_{24}N_3O_5S_2$, 474.1157.

The ^{1}H NMR and ^{13}C NMR spectra of new compounds

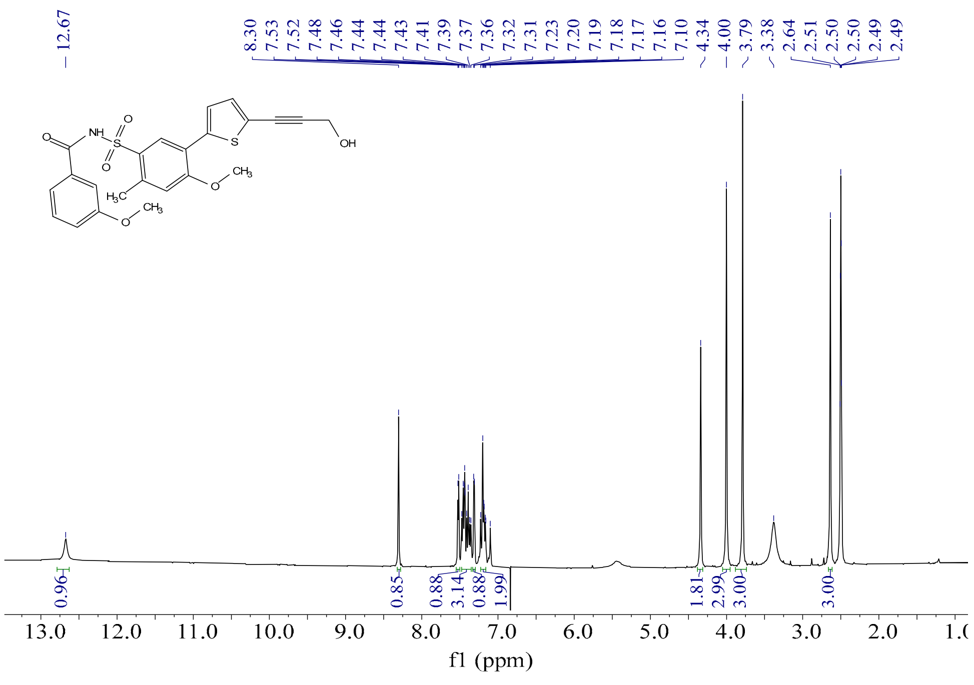

Figure S5. ^{1}H NMR (400 MHz, DMSO-d_6) of **SW-a**

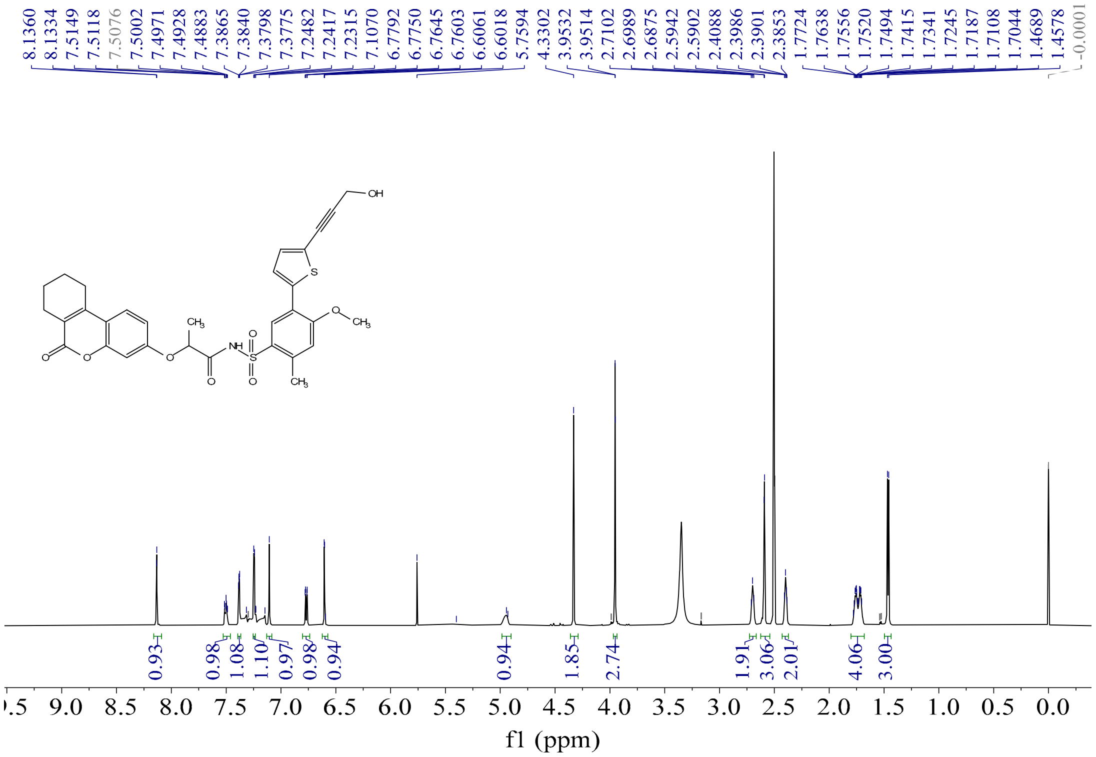

Figure S6. ^{1}H NMR (600 MHz, DMSO-d_6) of **SW-b**

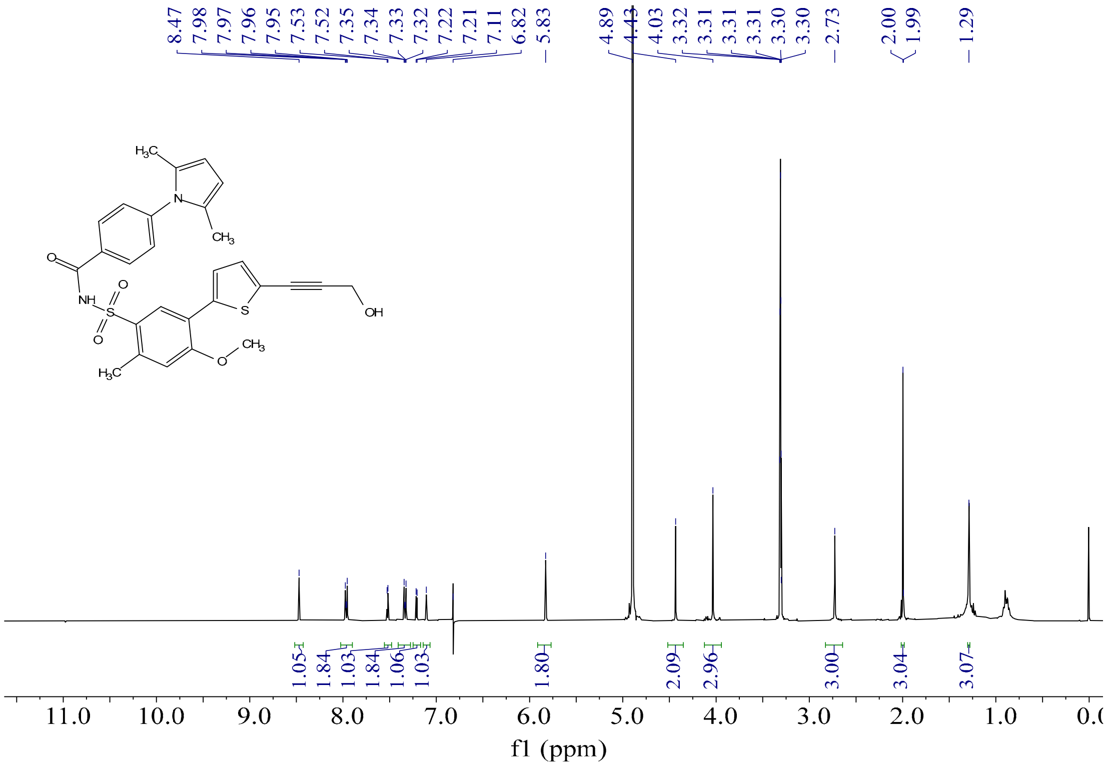

Figure S7. ^{1}H NMR (400 MHz, Methanol-d_4) of **SW-c**

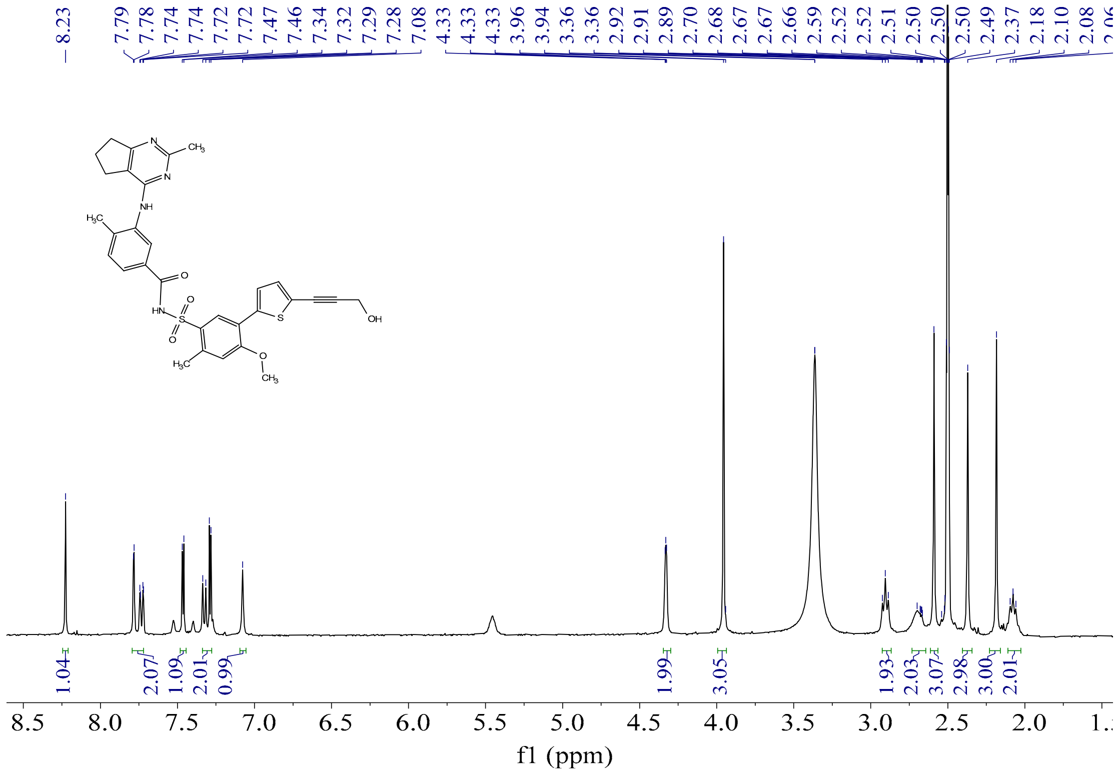

Figure S8. ^{1}H NMR (400 MHz, DMSO-d_6) of **SW-d**

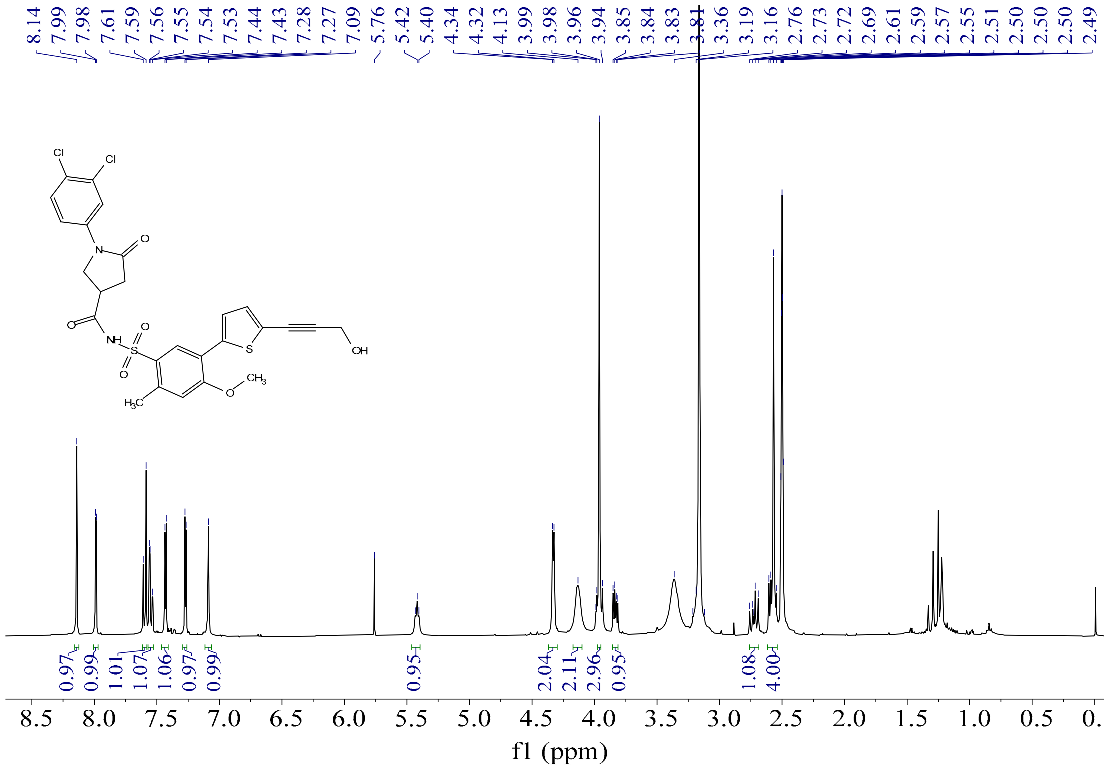

Figure S9. ^{1}H NMR (400 MHz, DMSO-d_6) of **SW-e**

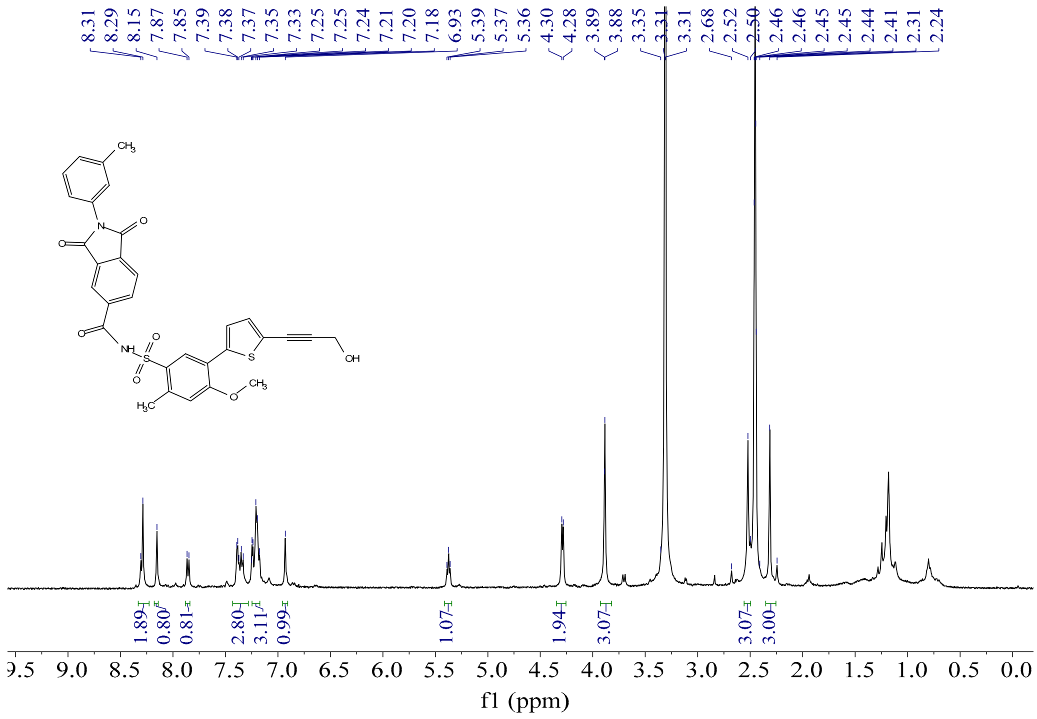

Figure S10. ^{1}H NMR (400 MHz, DMSO-d_6) of **SW-f**

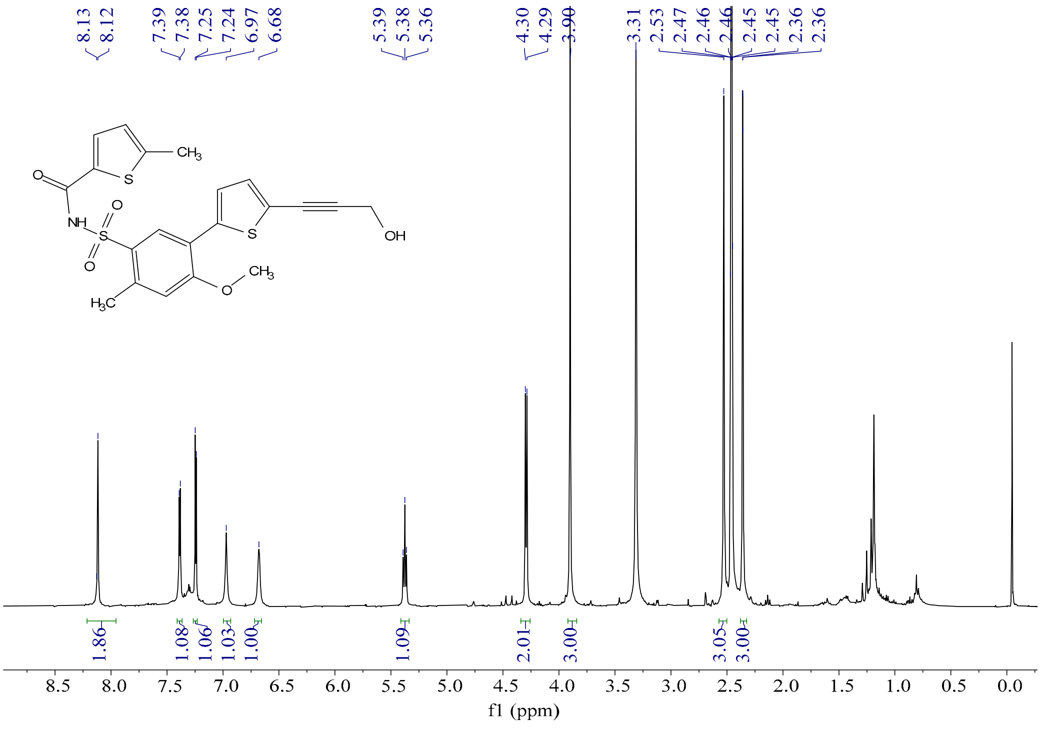

Figure S11. ^{1}H NMR (400 MHz, DMSO-d_6) of **SW-g**

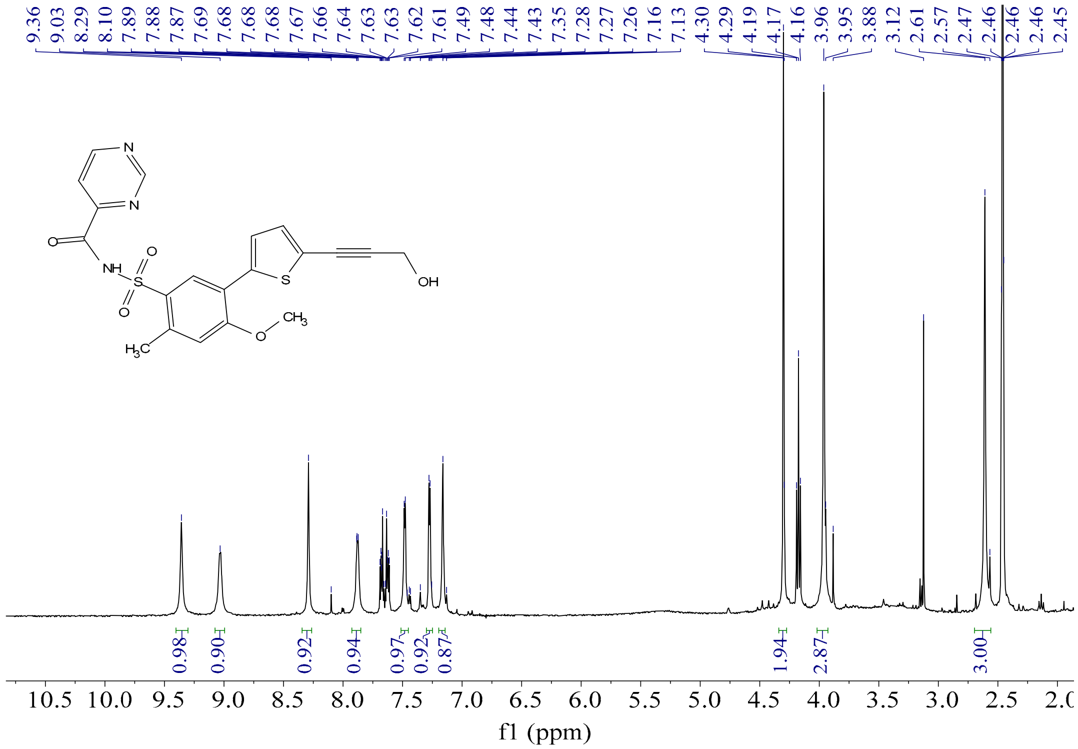

Figure S12. ^{1}H NMR (400 MHz, DMSO-d_6) of **SW-h**

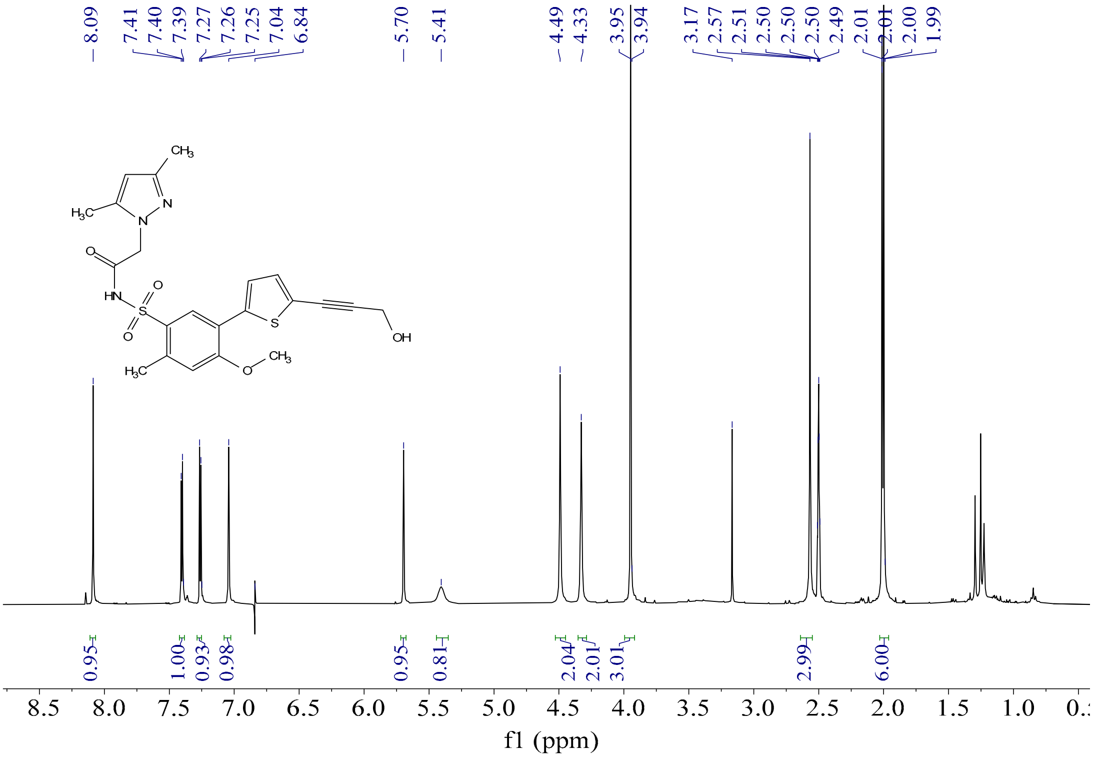

Figure S13. ^{1}H NMR (400 MHz, DMSO-d_6) of **SW-i**

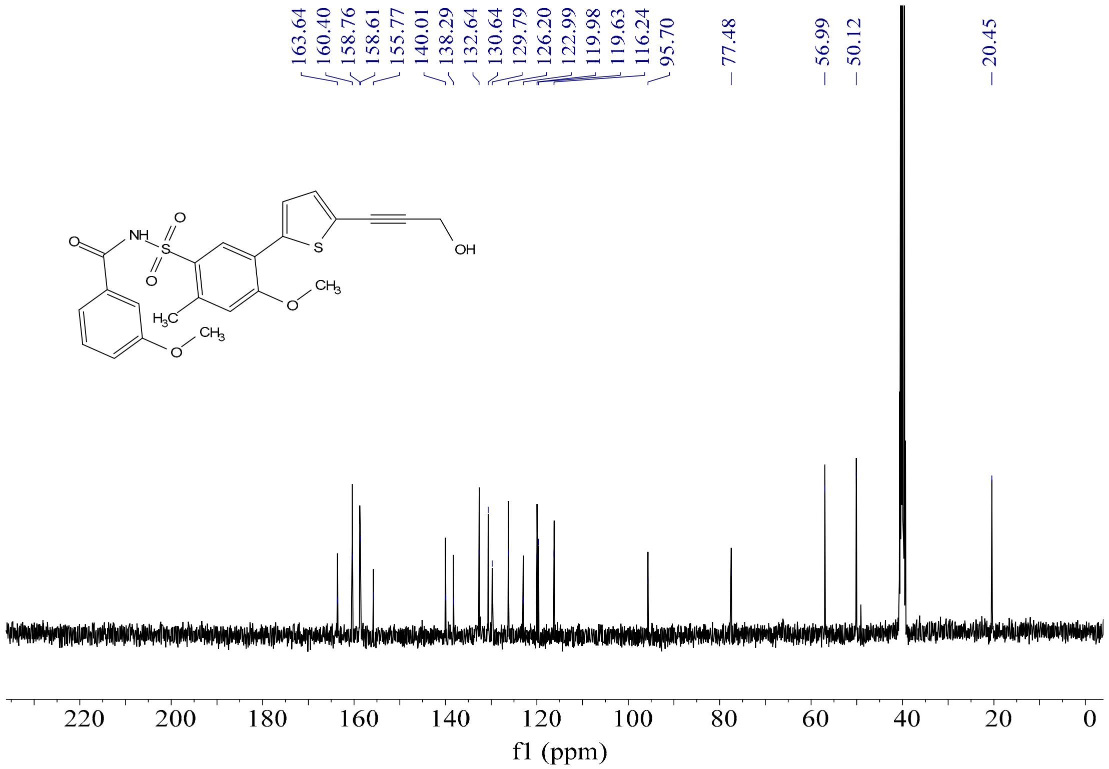

Figure S14. ^{13}C NMR (101 MHz, DMSO-*d*$_6$) of **SW-a**

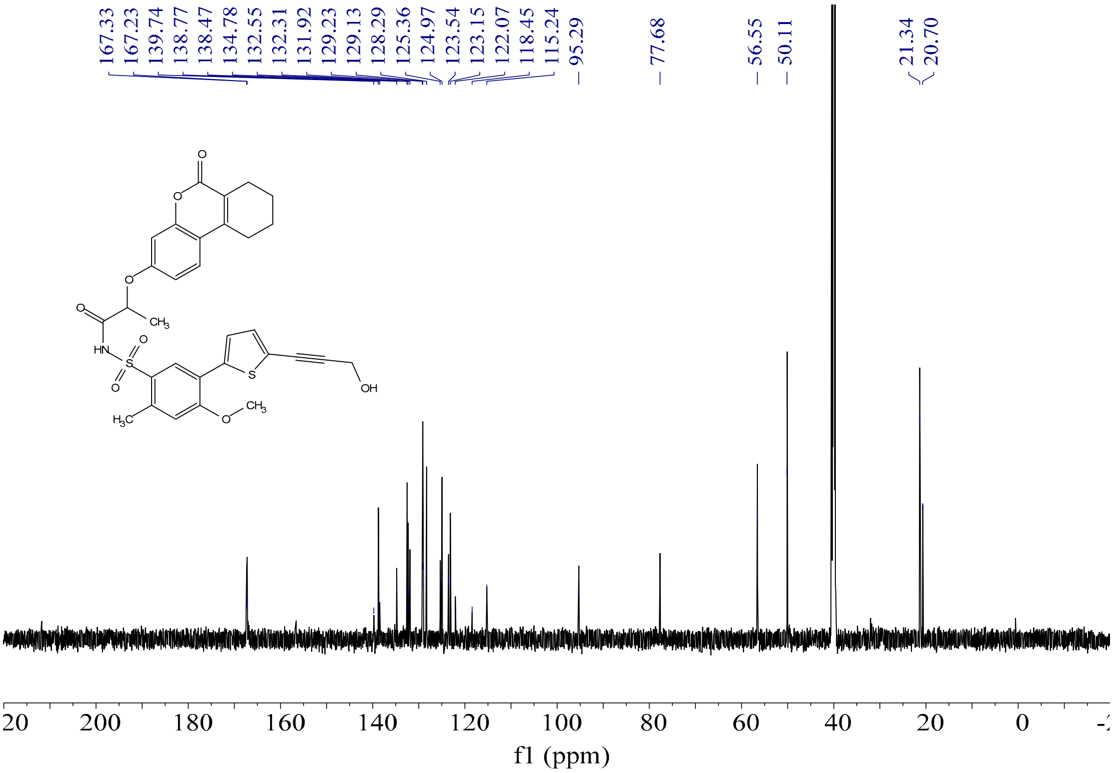

Figure S15. ^{13}C NMR (101 MHz, DMSO-*d*$_6$) of **SW-b**

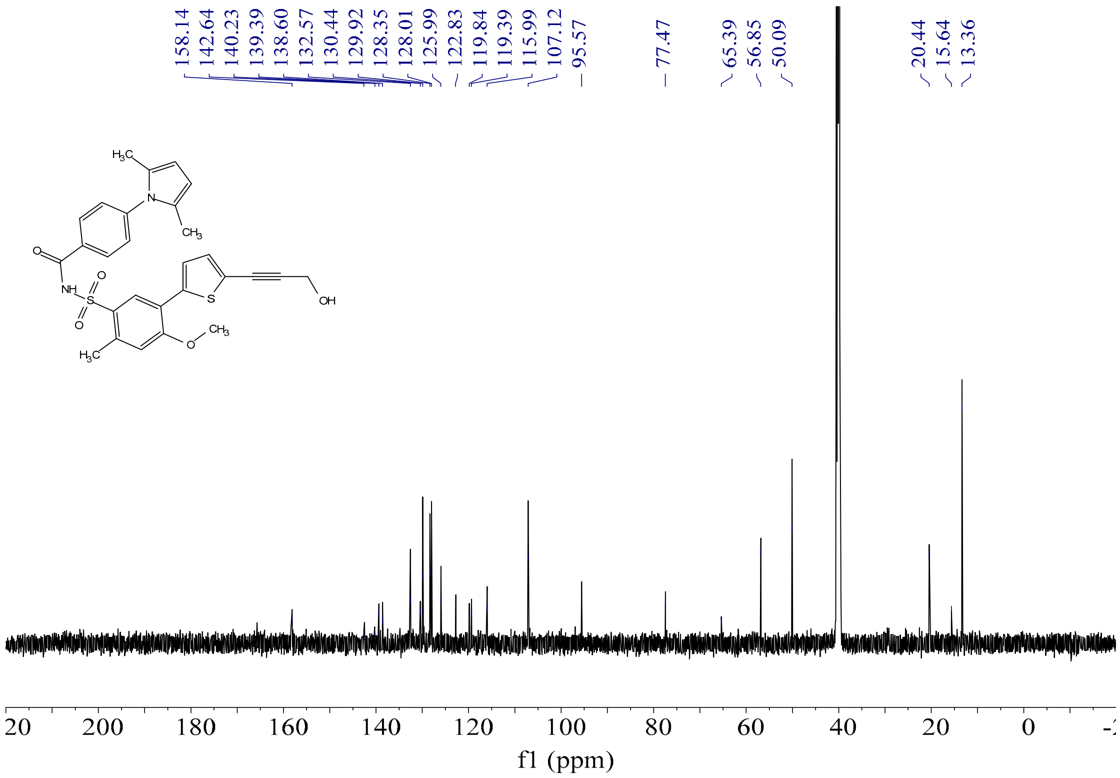

Figure S16. ^{113}C NMR (400 MHz, DMSO-d_6) of **SW-c**

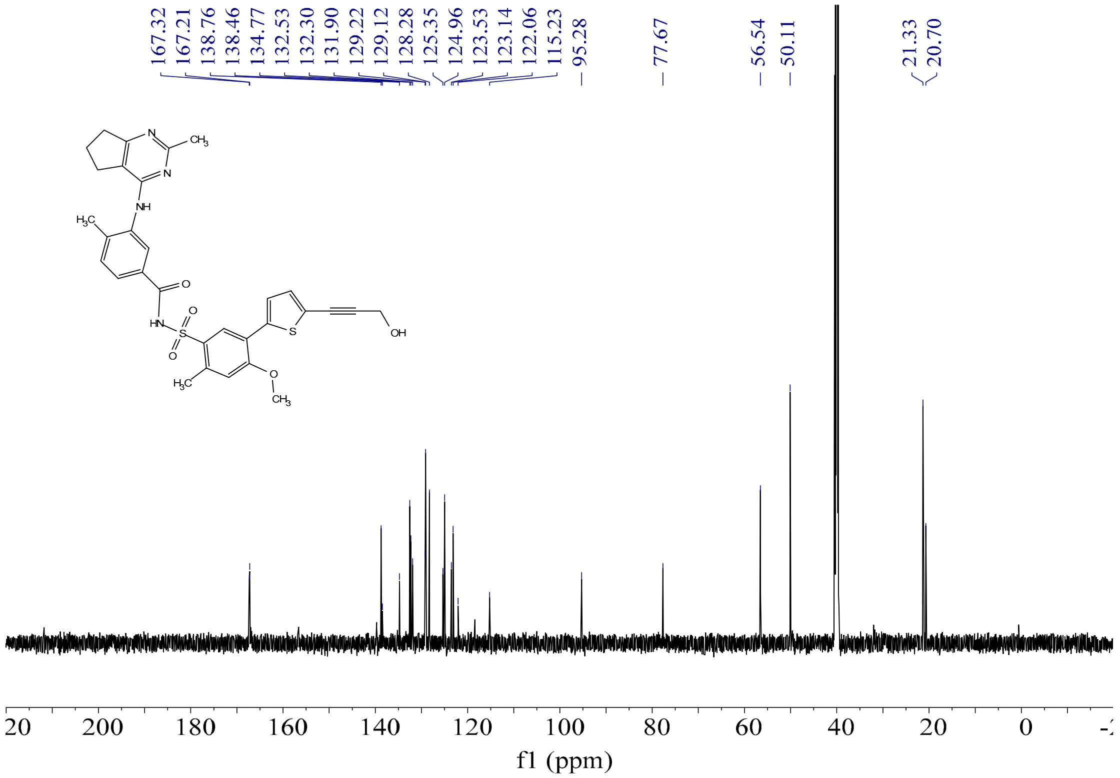

Figure S17. ^{13}C NMR (101 MHz, DMSO-d_6) of **SW-d**

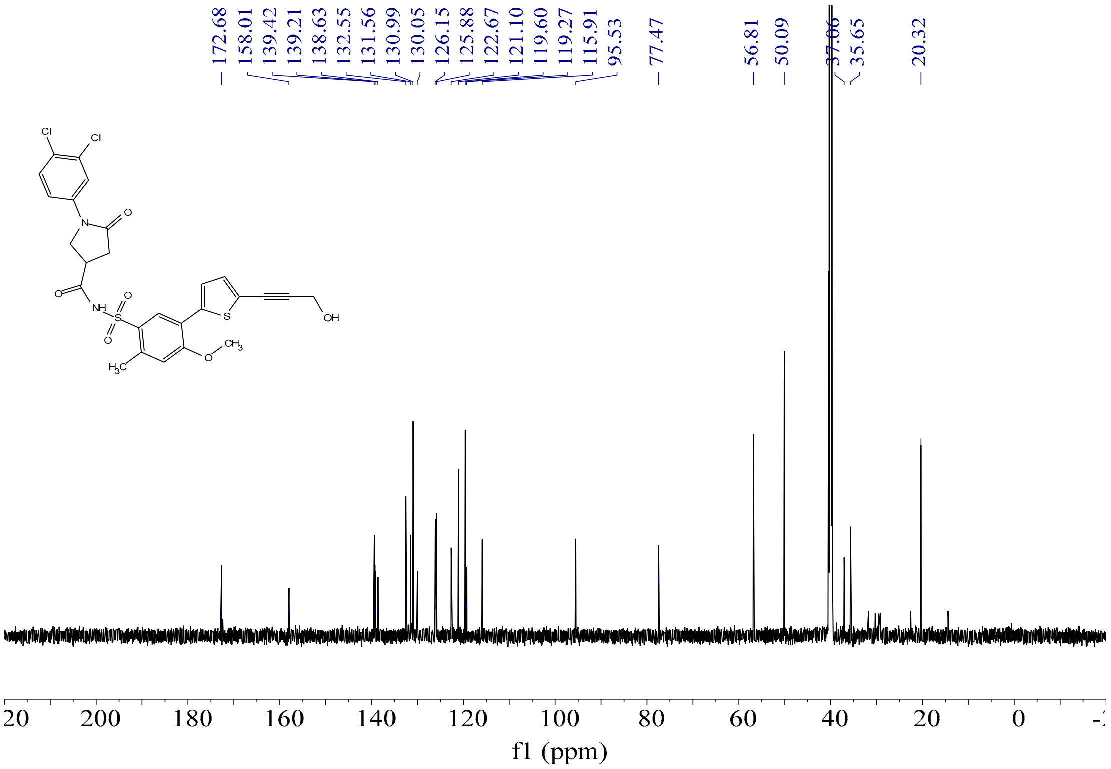

Figure S18. ^{13}C NMR (101 MHz, DMSO-d_6) of **SW-e**

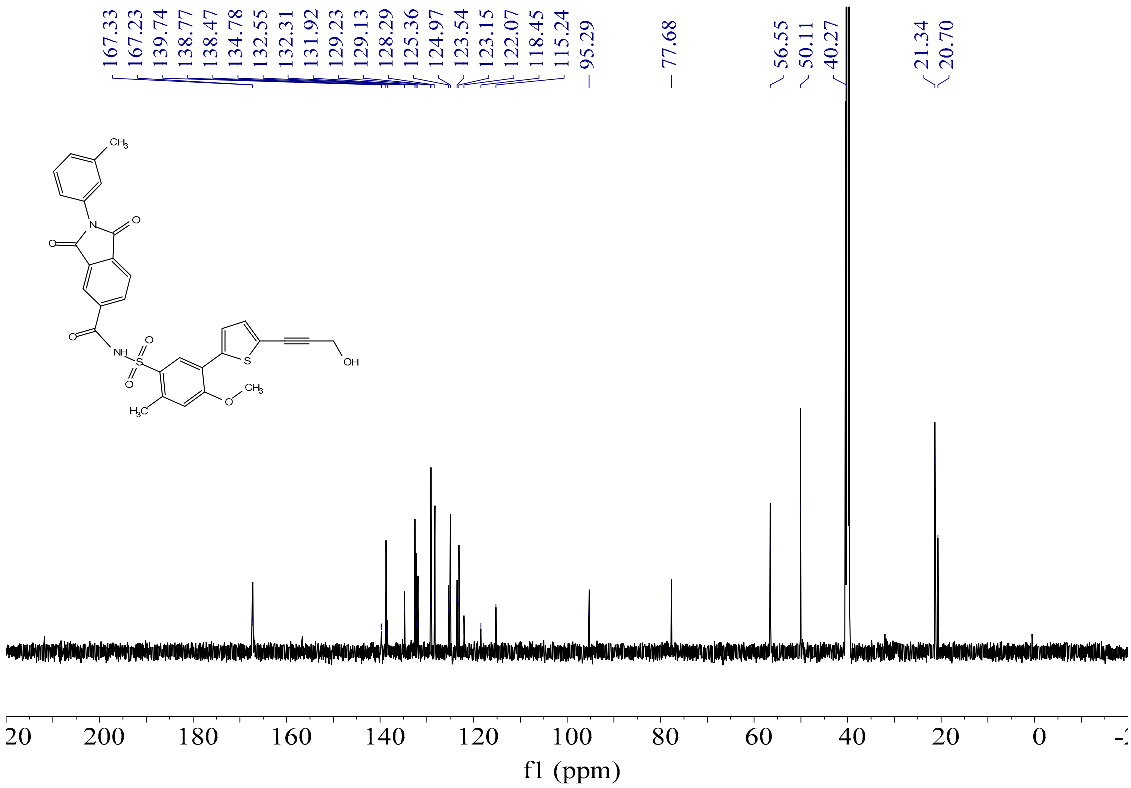

Figure S19. ^{13}C NMR (101 MHz, DMSO-d_6) of **SW-f**

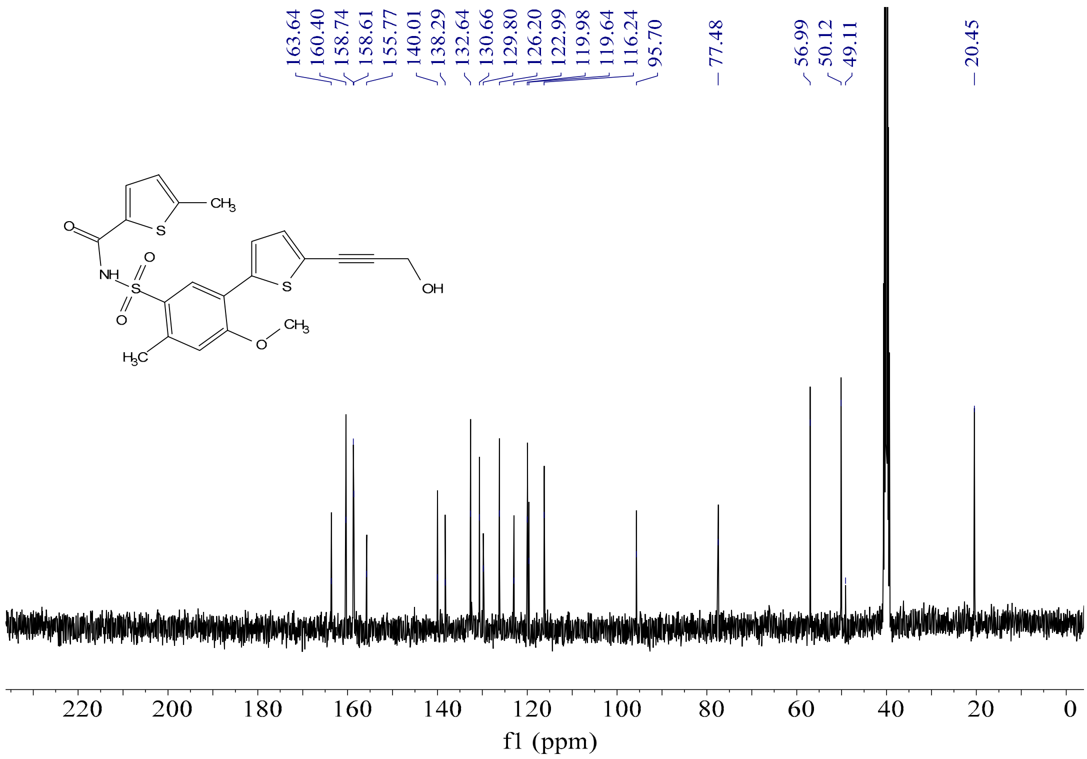

Figure S20. ^{13}C NMR (101 MHz, DMSO-d_6) of **SW-g**

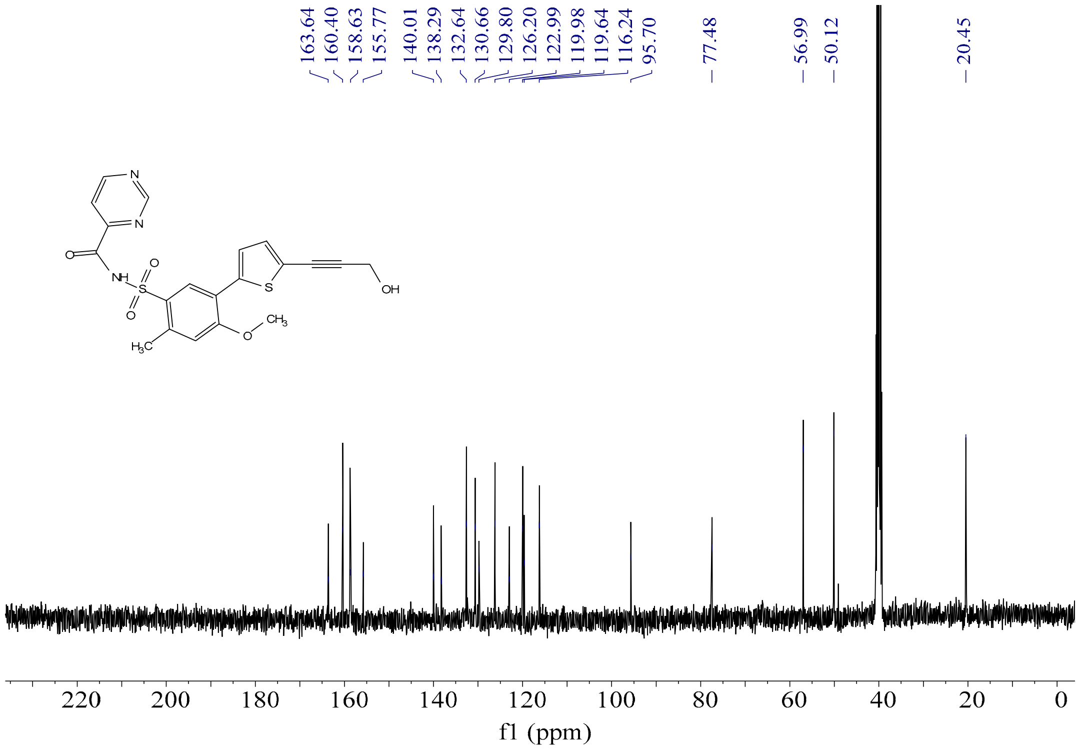

Figure S21. ^{13}C NMR (101 MHz, DMSO-d_6) of **SW-h**

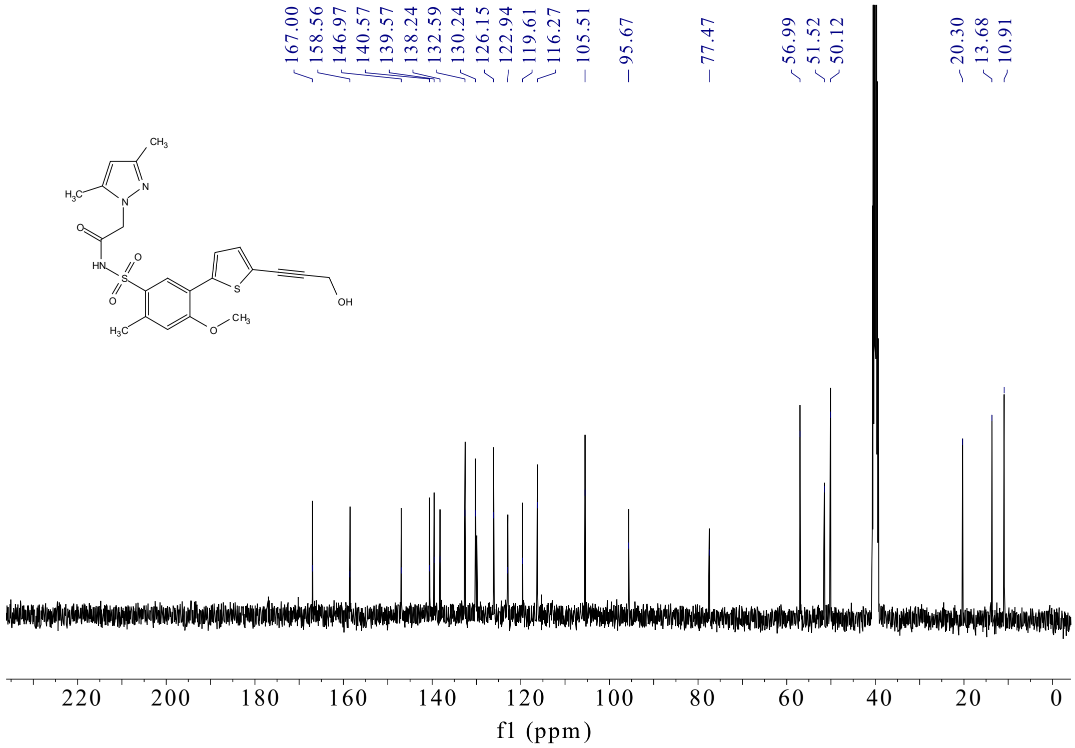

Figure S22.^{13}C NMR (101 MHz, DMSO-d_6) of **SW-i**

The MS spectra of new compounds

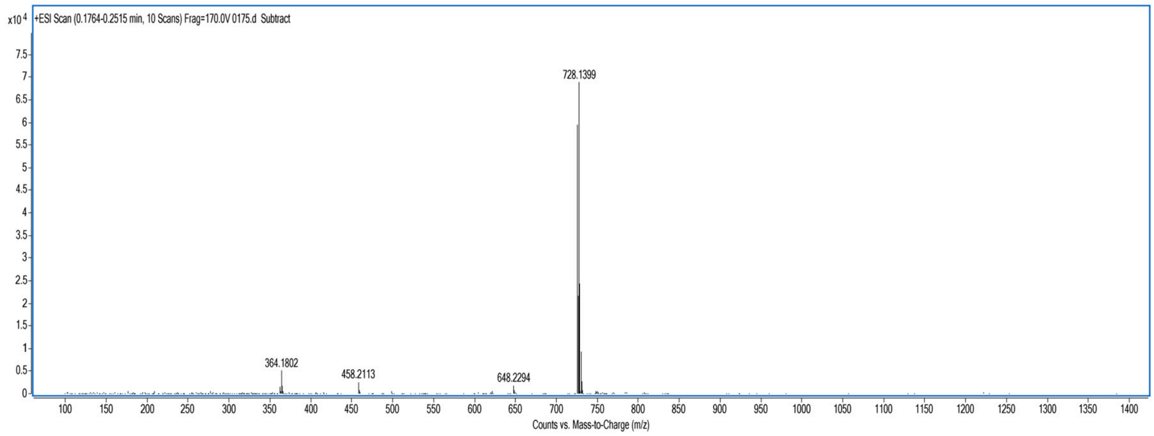

Figure S23. MS spectra of **SW-a**

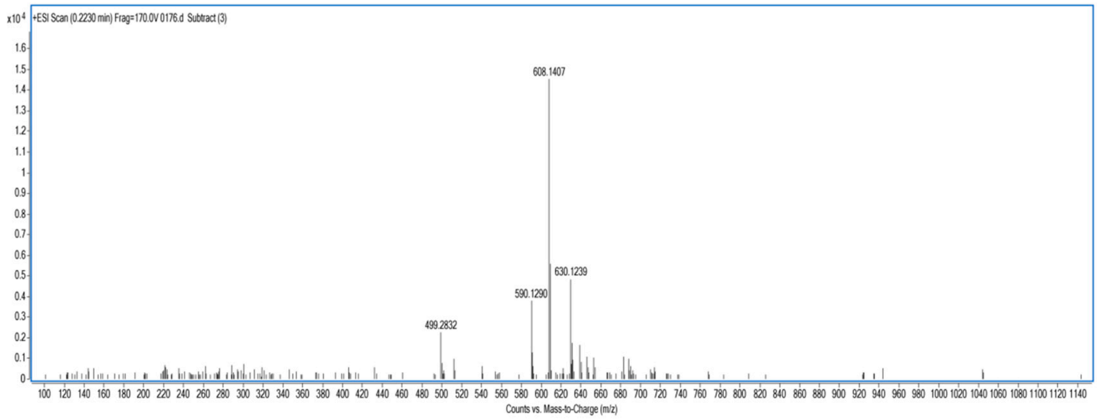

Figure S24. MS spectra of **SW-b**

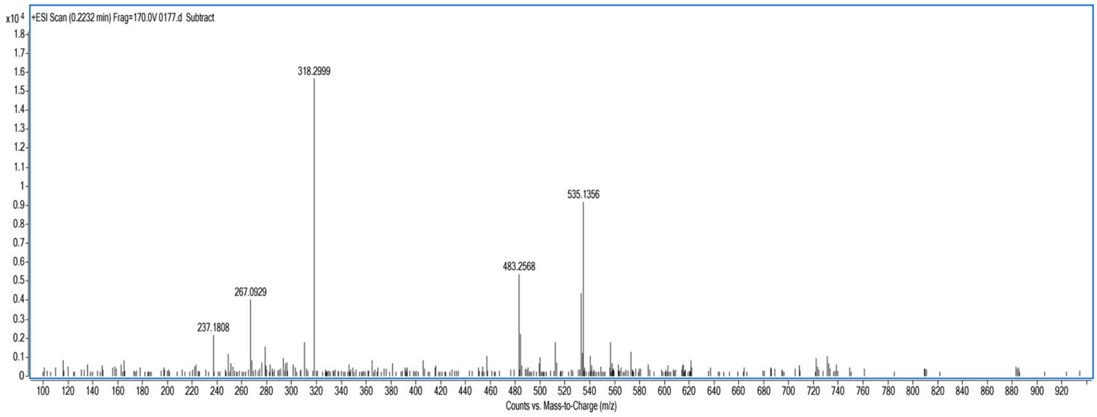

Figure S25. MS spectra of **SW-c**

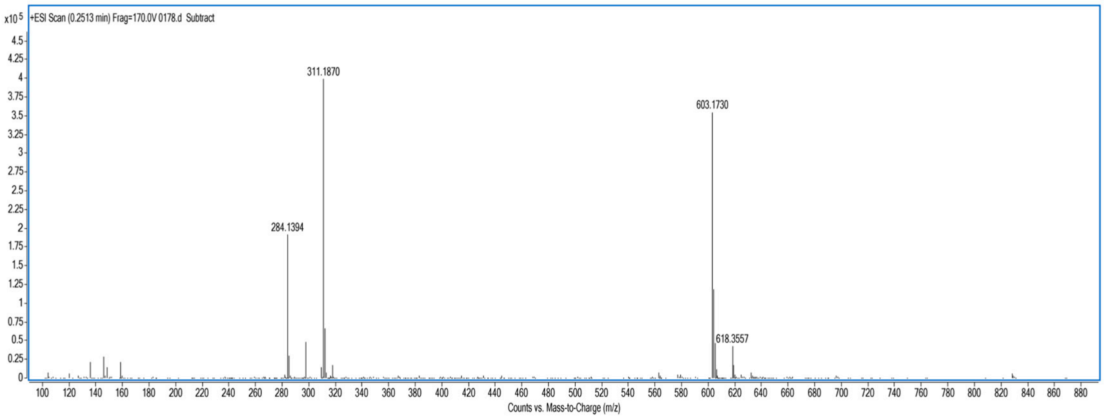

Figure S26. MS spectra of **SW-d**

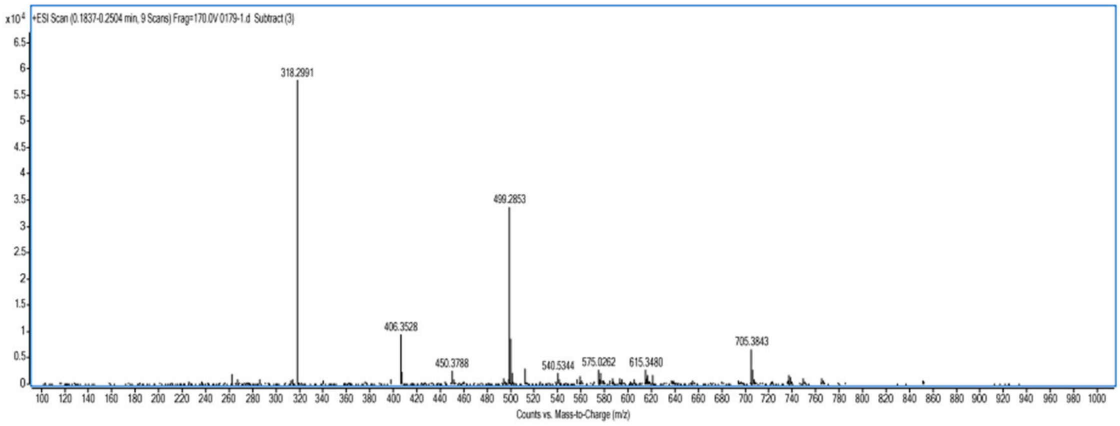

Figure S27. MS spectra of **SW-e**

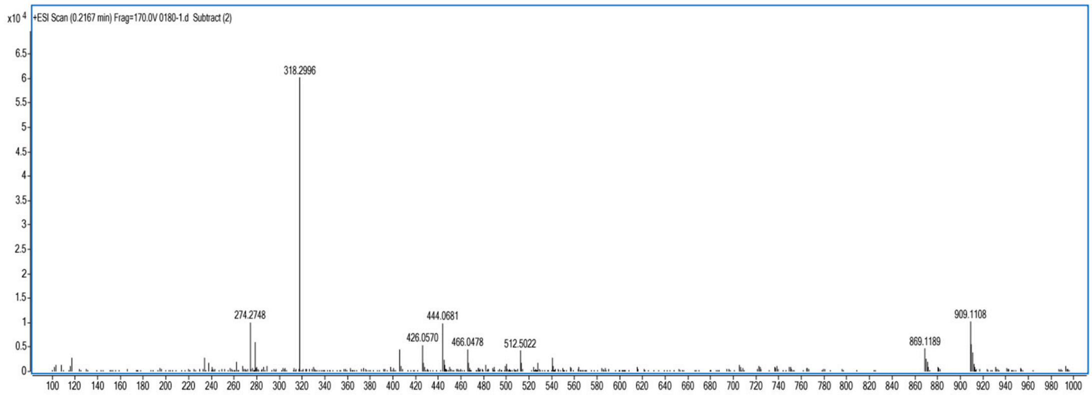

Figure S28. MS spectra of **SW-h**

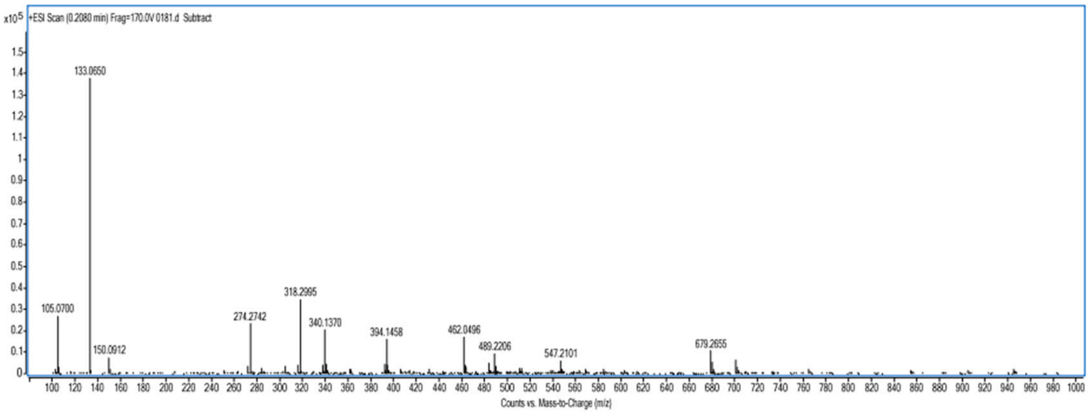

Figure S29. MS spectra of **SW-g**

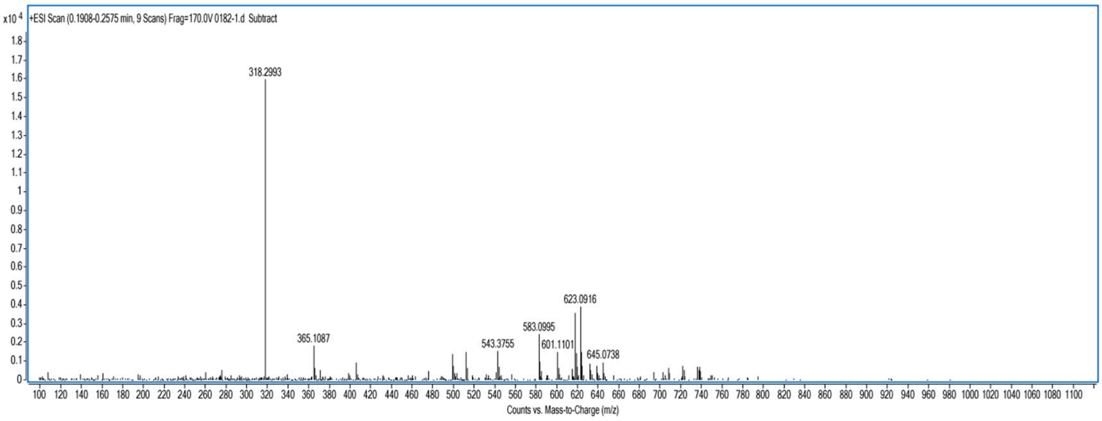

Figure S30. MS spectra of **SW-f**

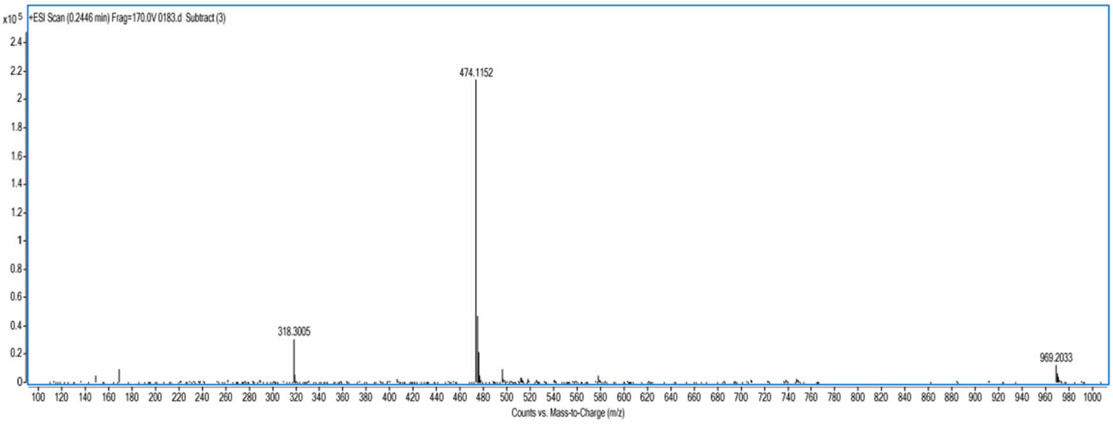

Figure S31. MS spectra of **SW-i**

The HPLC date and method

The purity of each synthesized compound was determined using reversed-phase high-performance liquid chromatography (HPLC). A 4.6-mm inside diameter (i.d.) by 250-mm length, 3.0-μm-particle size Agilent Pursuit XRs diphenyl column was employed. The column was eluted at a flow rate of 1 mL/min at 25 °C, and the absorbance was monitored at 254 nm using a gradient elution with water and MeOH. The elution process followed the following steps: 0–5 min, 0–100% MeOH; 5–30 min, 100% MeOH.

#	[min]		[min]	[mAU*s]	[mAU]	%
1	3.156	BB	0.1596	38.11094	3.54011	0.1301
2	3.647	BB	0.1171	30.02633	3.46486	0.1025
3	4.814	BB	0.1009	17.66853	2.68203	0.0603
4	7.750	VB R	0.0902	37.63733	6.07851	0.1285
5	9.529	BB	0.1252	2.90366e4	3712.98950	99.1127
6	10.852	BB	0.1012	7.95298	1.14278	0.0271
7	13.068	BB	0.0946	22.80579	3.66662	0.0778
8	15.906	BB	0.0959	70.75937	11.16548	0.2415
9	19.459	BB	0.1218	15.64510	1.94576	0.0534
10	25.947	BV	0.1288	10.98803	1.20000	0.0375
11	26.065	VB	0.1067	8.34412	1.12270	0.0285

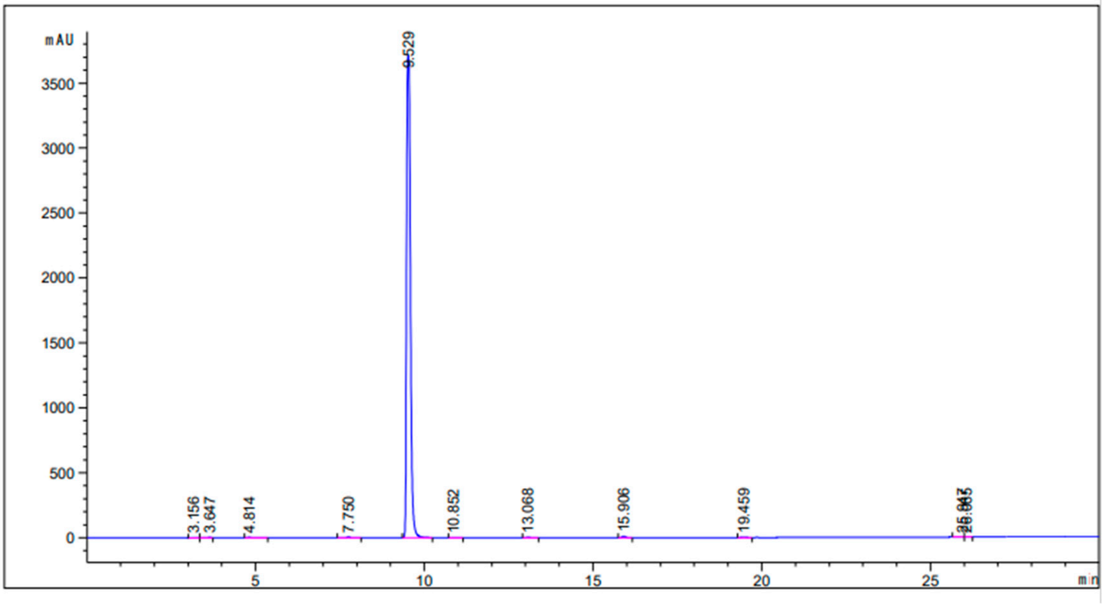

Figure S32. The HPLC date of compound **SW-a**
